# Association between the COVID-19 pandemic and childhood development aged 30 to 36 months in South Korea, based on the National health screening program for infants and children database

**DOI:** 10.1186/s12889-024-18361-9

**Published:** 2024-04-09

**Authors:** Kyung-Shin Lee, Youn Young Choi, You Sun Kim, Yeonjae Kim, Myoung-Hee Kim, Nami Lee

**Affiliations:** 1 Public Health Research Institute, National Medical Center, 245, Eulji-ro, Jung-gu, 04564 Seoul, Korea; 2 Department of Pediatrics, National Medical Center, 04564 Seoul, Korea; 3 Department of Pediatrics, Seoul National University Hospital, 03080 Seoul, Korea; 4 Division of Infectious Diseases, Department of Internal Medicine, National Medical Center, 04564 Seoul, Korea; 5 Center for Public Health Data Analytics, National Medical Center, 04564 Seoul, Korea; 6 Human Rights Center, Seoul National University Hospital, 03080 Seoul, Korea

**Keywords:** COVID-19, Children, Communication, Neurodevelopmental delay, Socioeconomic status

## Abstract

**Background:**

The coronavirus disease 2019 (COVID-19) pandemic has had a significant impact on the neurodevelopment of children. However, the precise effects of the virus and the social consequences of the pandemic on pediatric neurodevelopment are not yet fully understood. We aimed to compare the neurodevelopment of children between before and during the COVID-19 pandemic, as well as examine the impact of socioeconomic status (SES) and regional differences on the development.

**Methods:**

The study used the Korean Developmental Screening Test to compare the difference in the risk of neurodevelopmental delay between before and during the COVID-19 pandemic. Multivariable logistic regression analysis was conducted to identify the relationship between experiencing the COVID-19 pandemic and the risk of neurodevelopmental delay. Stratified analyses were performed to determine whether the developmental delays caused by the pandemic’s impact varied depending on SES or regional inequality.

**Results:**

This study found an association between the experience of COVID-19 and a higher risk of neurodevelopmental delay in communication (adjusted OR [aOR]: 1.21, 95% confidence interval [CI]: 1.19, 1.22; *P*-value: < 0.0001) and social interaction (aOR: 1.15, 95% CI: 1.13, 1.17; *P*-value: < 0.0001) domains among children of 30–36 months’ ages. Notably, the observed association in the Medicaid group of children indicates a higher risk of neurodevelopmental delay compared to those in the non-Medicaid group.

**Conclusions:**

These findings highlight the need to be concerned about the neurodevelopment of children who experienced the COVID-19 pandemic. The study also calls for increased training and support for Medicaid children, parents, teachers, and healthcare practitioners. Additionally, policy programs focused on groups vulnerable to developmental delays are required.

**Supplementary Information:**

The online version contains supplementary material available at 10.1186/s12889-024-18361-9.

## Background

On March 11, 2020, the World Health Organization (WHO) declared coronavirus disease 2019 (COVID-19) a pandemic [1]. By August 2, 2023, there had been 769 million confirmed cases of COVID-19, including 7 million who died from it [2]. In South Korea, the proportions of pediatric cases ≤ 19 years and ≤ 9 years were 21.8% and 9.5% of all confirmed cases, respectively, according to data from the Korea Centers for Disease Control and Prevention (KCDC) [3]. Additionally, the SARS-CoV-2 virus has caused 5,388,338 cases and 46 fatalities in children and adolescents who are ≤ 18 years of age as of September 3, 2022 [4].

The rapid spread of COVID-19 prompted countries worldwide to implement measures for reducing mortality, such as social isolation, home quarantine, educational institution closures, and case isolation [5]. In South Korea, more than 75% of preschool children attended a daycare center or kindergarten [6], but had to face unusual educational challenges in these facilities for preschoolers during the COVID-19 pandemic [7]. The educational institutions were closed to decrease the risk of disease transmission within the institutions and during the journey to and from them, with face-to-face contact also limited during the pandemic owing to physical distance restrictions. Huang et al. reported that these containment policies limit children’s outdoor activities and opportunities to interact with people during the pandemic in the Guangzhou Province of China, providing insights from a single-country experience [8]. Child neurodevelopment is linked to various biological and psychological factors, including maternal physical and mental health, physical exercise, socioeconomic level, and family setting [9, 10]. Experiencing the pandemic has raised anxiety and sadness in the general population [11], and parental and caregiver mental health difficulties may negatively impact child development [12].

However, few studies have investigated the association between neurodevelopment and the social aftermath of the COVID-19 pandemic. Huang et al. revealed that experiencing the COVID-19 pandemic and the public health strategies were associated with a higher risk of delay in developing fine motor and communication in 1-year-old children, which was reported as the first study to reveal the risk of delayed neurodevelopment [8]. They mentioned that the COVID-19 epidemic may have harmed the mental health of the children’s parents and decreased social contact due to the containment policy, thus affecting children’s language development. These findings imply that the COVID-19 pandemic may have a deleterious influence on child neurodevelopment in specific domains at specific ages, highlighting the development of young children during the pandemic [8]. Rapid brain growth occurs prenatally and throughout infancy until the age of 3 years; the timing of brain sensitivity for specific developmental domains varies significantly [13–15]. Additionally, health inequity based on socioeconomic status (SES) and geographic variation during the COVID-19 pandemic have been reported [16–19]. These studies suggested that the COVID-19 pandemic may disproportionately affect vulnerable children from low-income households and those living in disadvantaged locations. We aimed to compare children’s neurodevelopment between before and during the COVID-19 pandemic with respect to the impact of SES and regional differences.

## Methods

### Data source and study population

Since November 2007, the National Health Screening Program for Infants and Children (NHSPIC) in South Korea has been implemented nationwide to track the growth and development of infants and children and to provide appropriate education programs to caregivers. This includes the introduction of a health check-up program tailored to the age of infants and children [20]. The NHSPIC consists of seven screening rounds, which comprised physical examination, development evaluation including rounds 2 to 7, a general health questionnaire, and age-specific anticipatory counseling [21].

Developmental evaluation is the most important NHSPIC screening item, with the first-time examinations being performed at 9–12 months, the second at 18–24 months, the third at 30–36 months, the fourth at 42–48 months, the fifth at 54–60 months, and the sixth at 66–71 months [22]. Our study focused on children who underwent the third round of the NHSPIC, typically conducted between 30 and 36 months. In South Korea, social distancing measures were implemented on February 29, 2020, escalating to a “high level” on March 22, 2020. Subsequently, these measures were eased to a “moderate level” on April 20, 2020, with further relaxation occurring on May 6, 2020 [23]. Schools and kindergartens in the Seoul area, encompassing approximately one-fifth of the South Korean population, experienced closures from February 24, 2020, to May 20, 2020; December 23, 2020, to January 29, 2021; and July 12, 2021, to August 2, 2021, affecting over 600,000 enrolled children [24, 25]. Owing to the COVID-19 pandemic, the government temporarily extended the check-up window by 1–2 months for children who had not yet undergone evaluation until the subsequent round of assessments. We categorized children who underwent the NHSPIC examination from April 2018 to December 2019 as the “before pandemic group” and children who participated from April 2020 to December 2021 as the “pandemic group.” We checked the Korean Statistical Information Service data to assess if there was a change in the participant ratio before or during the COVID-19 period and SES level [26].

### Assessment of child neurodevelopment

The Korean Developmental Screening Test (K-DST) is a validated and reliable tool for developmental screening among Korean infants and children aged 4 to 71 months [20, 27]. The K-DST is a helpful evaluation tool for screening, developmental surveillance, and tracking post-treatment improvements in healthy children using six domains, including gross motor skills, fine motor skills, cognition, language, sociality, and self-care [28]. It comprises 200 questionnaires, and the primary caregivers (parents, grandparents) and alternative caregivers report on whether their child can perform the task described. The questions in each domain are scored from 0 to 3 points. Based on the sum of the total score in each domain, the K-DST results were classified into four levels, namely “need for follow-up,” “recommendation for further evaluation,” “peer level,” and “fast level.” The outcome of interest was suspected neurodevelopmental delay, characterized by the results of “need for follow-up” or “recommendation for further evaluation” in each K-DST domain at 30–36 months of age [29]. If any of the six domains showed suspected neurodevelopmental delay, the “any domain” was defined as a suspected neurodevelopmental delay.

### Variables

We estimated 11 covariates that could affect neurodevelopment outcomes including sociodemographic (the level of residence, SES level, age [in months], and sex [boys/girls]), disability (Yes/No), health status (preterm birth [< 37 weeks of gestational age], or low birth weight at birth (birth weight < 2500 g]), maternal characteristics (maternal age group, mother’s nationality), health care accessibility (number of pediatricians per population of children in residence), and K-DST data-related variables ([delayed visit [yes/no], and survey interval type [three visits of interval such as 30–32, 33–35, and 36–41 months]). Information on a range of mother and child characteristics was obtained from questionnaires and medical records from the National Health Insurance Service (NHIS) data in South Korea. Maternal characteristics were collected from a national mother-child cohort (NMCC) [30] in the NHIS data, including the mother’s age (20–29, 30–39, and ≥ 40) and nationality (Korean or foreign). If participants were not included in the NMCC, we categorized the maternal information as ‘unknown.’ For the stratification variable, we defined SES levels as follows: a child covered by Medicaid was classified into the Medicaid group, while those with insurance coverage or whose parent was a self-employed employee were categorized into the non-Medicaid group. In 2022, out of 52.93 million insurers, 51.41 million were beneficiaries of health insurance and 1.52 million were beneficiaries of Medicaid. We also classified residential areas as urban for children residing in nine major cities and rural for children residing in nine provinces. If a child’s age at the third-round visit ranged from 37 to 41 months, the visit was classified as delayed.

### Statistical analysis

Descriptive statistics comprised frequencies, means, and standard deviations of the characteristics of the children, both before and during the COVID-19 pandemic period. The proportions of neurodevelopmental delay in each domain were presented for both periods, and a chi-square test was conducted to compare the proportions before and during the COVID-19 pandemic. Univariate logistic regression analysis was employed to calculate the odds ratios (ORs) and 95% confidence intervals (CI) for the relationship between experiencing the COVID-19 pandemic and the risk of neurodevelopmental delay in each section. After accounting for all confounding variables, including children’s sex, age (continuous), urban, preterm, low birth weight, maternal age group, mother’s nationality, number of pediatricians per population of children, delayed visits, and survey section type, we utilized a multivariable logistic regression analysis to identify the relationship within these associations. With two-sided alpha-level adjusting for multiple comparisons (Bonferroni correction *p* < 0.001), the study was performed while considering the potential for Type I errors.

To assess whether the developmental delays caused by the impact of the COVID-19 pandemic varied depending on SES and regional inequality, we performed stratified analyses exploring the association between experiencing the pandemic and neurodevelopmental outcomes according to the SES level and residence. The non-Medicaid group was stratified into two separate subgroups, i.e., self-employed individuals and insured employees. The non-Medicaid group was stratified into five categories based on a combination of the income quintile and insurance type. By comparing the risk of developmental delay among these strata, we aimed to gain a more nuanced understanding of the potential role of SES in this context. We visualized the difference in adjusted ORs using the *‘ggplot2’* function in the R package in supplementary figure S2 and S3 [29]. A two-sided *P* < 0.05 was considered statistically significant. All analyses were conducted using SAS software (version 9.4; SAS Institute, Cary, NC, USA).

### Ethics statement

This study was approved by the Institutional Review Board of the National Medical Center (approval number: NMC-2023-01-001) and performed according to the tenets of the Declaration of Helsinki. Owing to the retrospective nature of this study, the requirement for informed consent was waived.

## Results

### Descriptive characteristics

**Fig. 1:**
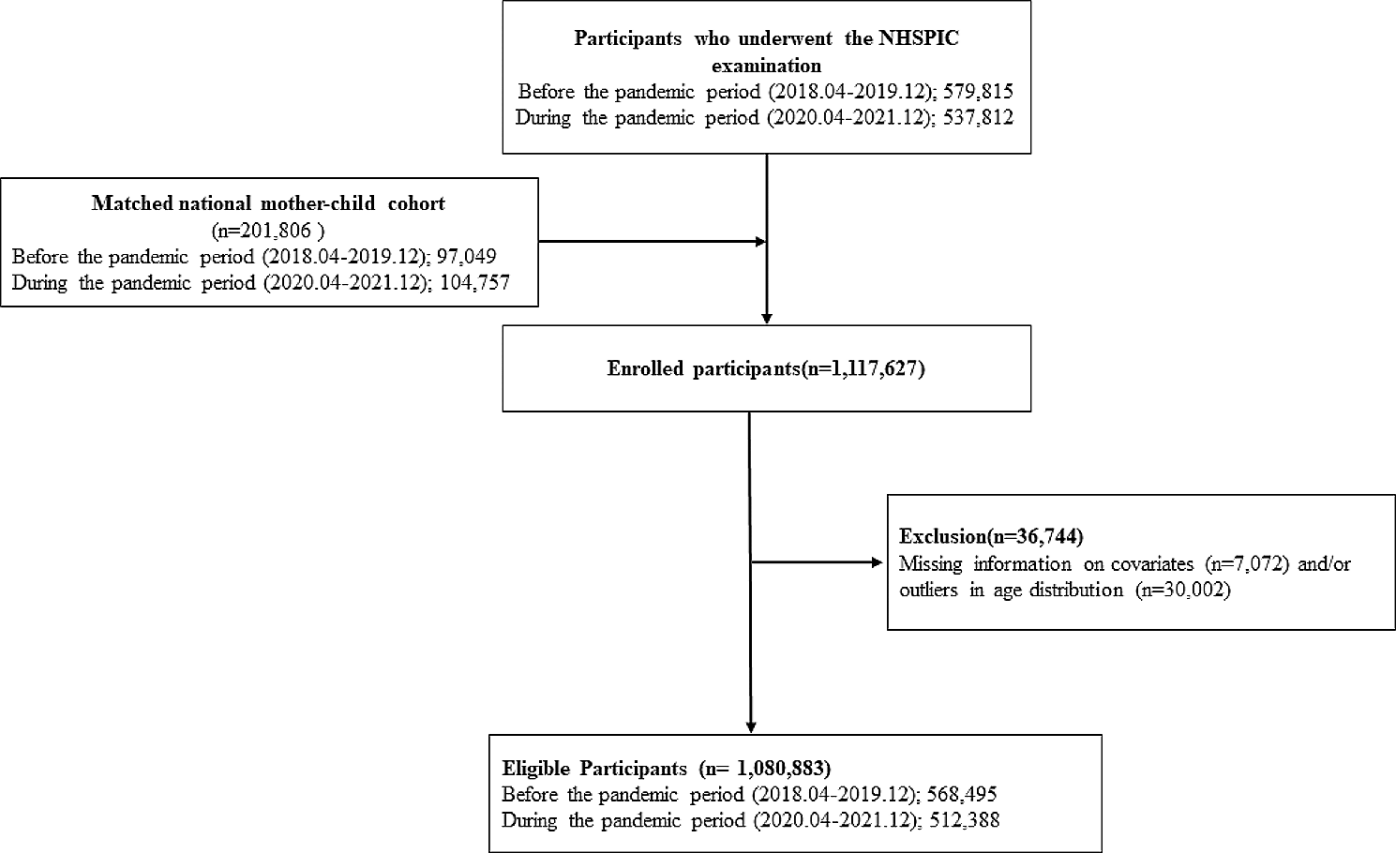
Diagram of the study

We excluded cases with missing data on covariates, including the children’s sex (n = 323), number of pediatricians in residence (n = 6,746), survey section type (n = 3), and age outliers (n = 30,002). Accordingly, 1,080,883 children were included in the analysis for the 30–36-months follow-up. Specifically, the neurodevelopmental screening data of 568,495 and 512,388 children from the before and during the COVID-19 pandemic were collected, respectively, as shown in Fig. 1. The participants’ characteristics are presented in Table 1. Overall, 48.75%, 4.68%, 1.05%, and 0.26% of the “before the pandemic group” comprised girls, children born preterm or with LBW, children from the Medicaid group, and children with disabilities, respectively. Additionally, 18.13% of children had delayed visits to K-DST. Children born to mothers aged 40 and over accounted for 11.26%, while children from mothers having Korean nationality accounted for 80.28%. The “during pandemic group” comprised 9.4% of children born preterm or LBW, 0.96% of those from the Medicaid group, and 0.31% with disabilities. Notably, 33.54% of visits were delayed beyond the recommended age of 30–36 months for developmental screening tests, a higher proportion than observed before the pandemic.

**Table 1:** Basic characteristics of the participants among 30–36 months old

	Before COVID-19 pandemic (2018.04-2019.12)	During COVID-19 pandemic (2020.04-2021.12)	*P*-value
	N(%)	N(%)	
Total	568,495	512,388	
Age(continuous)	34.45 ± 2.20	35.34 ± 2.51	< 0.0001
Preterm birth or LBW			
Yes	26,585 (4.68)	48,149 (9.4)	< 0.0001
No	541,910 (95.32)	464,239 (90.6)	
Sex			
Boys	291,337 (51.25)	262,957 (51.32)	0.449
Girls	277,158 (48.75)	249,431 (48.68)	
Types of the insured ^a^			
Medicaid	5,993 (1.05)	4,942 (0.96)	< 0.0001
Non-Medicaid	562,502 (98.95)	507,446 (99.04)	
Disability			
None	567,042 (99.74)	510,808 (99.69)	< 0.0001
Yes (Mild or Severe)	1,453 (0.26)	1,580 (0.31)	
Number of pediatricians per population of children			
None	11,858 (2.09)	11,032 (2.15)	< 0.0001
Under mean	270,451 (47.57)	234,100 (45.69)	
Mean and over	286,186 (50.34)	267,256 (52.16)	
Region			
Urban	240,852 (42.37)	211,700 (41.32)	< 0.0001
Rural	327,643 (57.63)	300,688 (58.68)	
Delayed visit			
No	465,405 (81.87)	340,555 (66.46)	0.0001
Yes	103,090 (18.13)	171,833 (33.54)	
Section type of survey			
For 30–32 months	159,372 (28.03)	105,735 (20.64)	< 0.0001
For 33–35 months	229,642 (40.39)	176,476 (34.44)	
For 36–41 months	179,481 (31.57)	230,177 (44.92)	
Mother’s age group			
20–29	41,273 (7.26)	33,349 (6.51)	< 0.0001
30–39	366,147 (64.41)	300,841 (58.71)	
40 and over	64,026 (11.26)	73,441 (14.33)	
Unknown	97,049 (17.07)	104,757 (20.44)	
Mother’s nationality			
Korea	456,407 (80.28)	391,861 (76.48)	< 0.0001
Foreign	17,387 (3.06)	18,183 (3.55)	
Unknown	94,701 (16.66)	102,344 (19.97)	

a Insured type of parents or legal guardians. Others encompass two types of insured individuals: the employee insured and the self-employed insured

Screening results for neurodevelopmental delays in each section before and during the COVID-19 pandemic are presented in Table 2. The number of children with suspected neurodevelopmental delay, determined by the any domain of the K-DST, was 92,045(16.19%) before the COVID-19 period and 87,967(17.17%) during the COVID-19 pandemic. Compared to the period of before the COVID-19 pandemic, significantly higher proportions of suspected neurodevelopmental delay were observed during the COVID-19 pandemic in the following domains: fine motor skill (from 6.86 to 7.19%), cognition (from 5.90 to 6.22%), communication (from 8.69to10.01%), social interaction (from 6.74 to 7.62%), and self-care (from 7.16 to7.64%), except for gross motor skill domain (from 4.24 to 4.07%). Additionally, we observed a consistent pattern in the rate of NHSPIC checkups at 30–36 months, categorized by the type of National Health Insurance subscriber, which is closely associated with socioeconomic status levels, from 2018 to 2021 (Figure S1).

**Table 2:** The proportion of screened for the risk of neurodevelopmental delay in each developmental section between before and during COVID-19 period among 30–36 months old of children

The 7 domains of the K-DST	Before COVID-19 pandemic (2018.04-2019.12)	During COVID-19 pandemic (2020.04-2021.12)	*P*-value
	N(%)	N(%)	
Any domain	92,045 (16.19)	87,967 (17.17)	< 0.0001
Gross Motor	24,105 (4.24)	20,840 (4.07)	< 0.0001
Fine Motor	38,973 (6.86)	36,831 (7.19)	< 0.0001
Cognition	33,566 (5.90)	31,862 (6.22)	< 0.0001
Communication	49,376 (8.69)	51,270 (10.01)	< 0.0001
Social Interaction	38,320 (6.74)	39,029 (7.62)	< 0.0001
Self-Care	40,714 (7.16)	39,133 (7.64)	< 0.0001

### The association between the experience of the COVID-19 pandemic and the risk of neurodevelopmental delay

The relationship between the pandemic period and the risk of neurodevelopmental delay is shown in Table 3. Experiencing the pandemic period was associated with a higher risk of delay in the fine motor (adjusted OR [aOR]: 1.08, 95% confidence interval [CI]: 1.06, 1.09), cognition (aOR: 1.10, 95% CI: 1.08, 1.11), communication (aOR: 1.21, 95% CI: 1.19, 1.22), social interaction (aOR: 1.15, 95% CI: 1.13, 1.17), and self- care (aOR: 1.14, 95% CI: 1.12, 1.16) domains among 30–36 months old children.

**Table 3:** Multivariable logistic regression models for screening of neurodevelopmental delay between before and during COVID-19 period among 30–36 months old

The 7 domains of the K-DST	Screened for neurodevelopmental delay			
	OR	*P*-value	aOR	*P*-value
Any domain	1.07 (1.06, 1.08)	< 0.0001	1.11 (1.10, 1.12)	< 0.0001*
Gross Motor	0.96 (0.94, 0.98)	< 0.0001	0.96 (0.94, 0.97)	< 0.0001*
Fine Motor	1.05 (1.04, 1.07)	< 0.0001	1.08 (1.06, 1.09)	< 0.0001*
Cognition	1.06 (1.04, 1.07)	< 0.0001	1.10 (1.08, 1.11)	< 0.0001*
Communication	1.17 (1.15, 1.18)	< 0.0001	1.21 (1.19, 1.22)	< 0.0001*
Social Interaction	1.14 (1.12, 1.16)	< 0.0001	1.15 (1.13, 1.17)	< 0.0001*
Self-Care	1.07 (1.06, 1.09)	< 0.0001	1.14 (1.12, 1.16)	< 0.0001*

Adjusting for children’s sex, age(continuous), urban, socioeconomic status, preterm birth or LBW, maternal age group, mother’s nationality, number of pediatricians per population of children, delayed visit, disability, and section type of survey

* Significant after Bonferroni correction for 17 risk factors (*p*-value < 0.003)

### Results of analyses stratified for SES and residential area

**Fig. 2:**
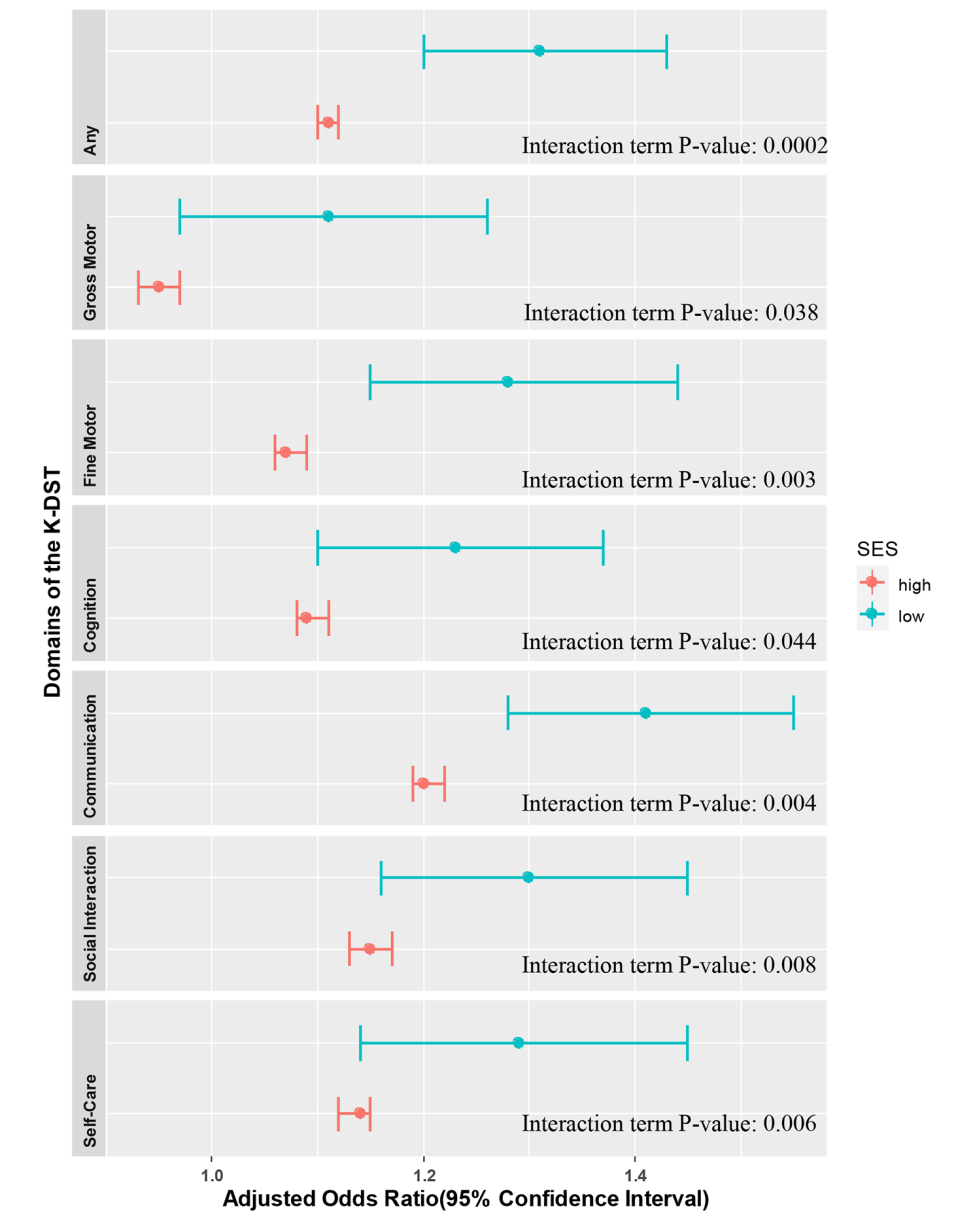
The associations between the experience of the COVID-19 pandemic and the risk of neurodevelopmental delay, stratified by SES level

**Fig. 3:**
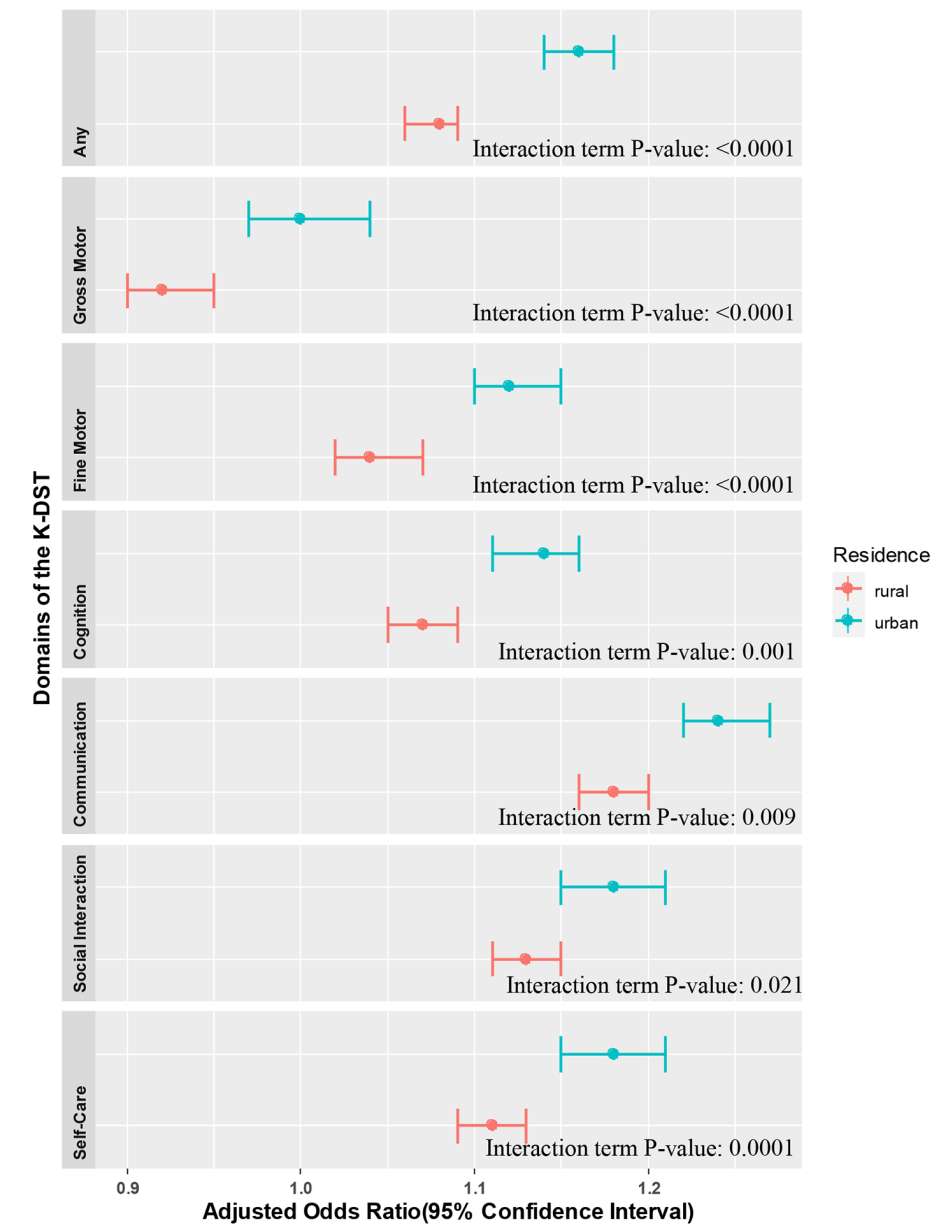
The associations between the experience of the COVID-19 pandemic and the risk of neurodevelopmental delay, stratified by level of residential area

Supplementary Tables S1 and S2 show the proportion of children with neurodevelopmental delay in the before and during COVID-19 period. Figure 2 and Table S3 display the associations between the experience of the COVID-19 pandemic and the risk of neurodevelopmental delay, stratified by SES level. Notably, among children in the Medicaid group, exposure to the COVID-19 pandemic was linked to higher aORs for suspected neurodevelopmental delay in all seven domains of the K-DST compared to the non-Medicaid group. Figure 3 and Table S6 show the association between the pandemic and the risk of neurodevelopmental delay, stratified by residential area (urban vs. rural). Residing in an urban area increased the risk of suspected neurodevelopmental delay in the total score and all six domains compared to rural areas. We also found that the risk of neurodevelopmental delay in the gross motor domain before the COVID-19 pandemic was higher than during the pandemic among children in the non-Medicaid group (aOR: 0.95, 95% CI: 0.93, 0.97) as well as those residing in rural areas (aOR: 0.92, 95% CI: 0.90, 0.95). To better understand the potential role of SES, we conducted a sensitivity analysis (Supplementary Tables S4 and S5); the results were consistent with those for the Medicaid and non-Medicaid groups (Supplementary Table S3).

### Sensitivity analysis

We conducted a sensitivity analysis based on the children’s age groups instead of the age-based survey interval type due to over-correction (Supplementary Table S7). Compared to the results presented in Table 3, the adjusted ORs for neurodevelopmental delay across all domains were lower in this analysis. However, the result of findings remained consistent, suggesting similar trends despite the differing levels of risk. Additionally, to address the potential impact of disabilities on neurodevelopmental outcomes, we have presented the results from analyses stratified for the presence of disabilities in Supplementary Table S8. The risk of developmental delay did not differ significantly between the before and during the COVID-19 periods in the disabled group, but increased significantly from before to during the COVID-19 period in the non-disabled group. Furthermore, to understand the effect of LBW, we conducted an analysis stratified by LBW and normal birth (Supplementary Table S9). Both the LBW and normal birth weight groups had an increased risk of developmental delay in the before and during COVID-19 period, but the adjusted OR for developmental delay was slightly higher in the LBW group. Supplementary Table S10 details the adjusted ORs for each covariate, facilitating an analysis of the potential risk factors for each domain of developmental delay. Although the adjusted ORs for developmental delay varied across domains, we found a consistent increase in the risk of developmental delay among children during the pandemic as compared to before the pandemic, with the following risk factors: male sex, Medicaid coverage, presence of disabilities, residence in areas with under the mean number of pediatricians, residence in urban areas, maternal age of 20–29 years, and foreign maternal nationality.

## Discussion

To our knowledge, this is the first nationwide study in Korea to reveal the impact of experiencing the COVID-19 pandemic on neurodevelopmental delay in children aged 30–36 months. Our findings revealed a significant association between experiencing the COVID-19 pandemic and a higher risk of neurodevelopmental delays in the communication and social interaction domains. Importantly, we observed that children’s neurodevelopmental delay among lower SES levels was associated with a higher risk than that among high SES levels. Additionally, children residing in urban areas had a slightly higher risk of suspected neurodevelopmental delay in the total score and in each domain than those in rural areas. The indirect consequences of the pandemic and lockdown measures, such as reduced or delayed medical services and school closures, have a disproportionately negative impact on children and adolescents [31]. Studies have consistently shown that experiencing the COVID-19 pandemic has a negative effect on the neurodevelopment of children, especially in communication and social interaction domains [8, 32]. These outcomes align with our own findings. Global closures of educational institutions have led to the highest percentage of children living in Asia among children not attending school during the COVID-19 pandemic, at 58% of 1.6 billion people [33]. With widespread closures of educational institutions such as kindergarten during the pandemic, children experienced learning setbacks and lost opportunities for social interaction with their peers, potentially leading to delays in neurodevelopment. Rao and Fisher noted that parents may have experienced increased stress due to job loss, income instability, and lack of patience with their children during the COVID-19 pandemic. These, in turn, could potentially have an adverse impact on their children’s neurodevelopment [32].

Huang et al. suggested that experiencing the pandemic in 2020 was associated with an increased risk of child developmental delay in the fine motor and communication domain among 1-year-old children; however, no such association was observed among children 6 months of age [8]. They reported that the opportunities for interactions with other family members, friends, and community members decreased during the COVID-19 pandemic due to measures such as school closure and social distancing. Consequently, this led to a negative impact on children’s language development [8]. In a systematic review and meta-analysis on the association of the COVID-19 pandemic during pregnancy with neurodevelopmental outcomes during infancy, risk of communication impairment and fine motor impairment, compared with their before pandemic counterparts, were reported [33]. Furthermore, the development of language and communication in children appears to be the most susceptible at the preschool age; this is consistent with the findings from a previous study by Huang and Hessami [8, 33]. Notably, we found that the COVID-19 pandemic had a positive impact on the children’s gross motor skills. This finding contrasts with results observed in older children in previous studies. Ayubi et al. reviewed the impact of the COVID-19 pandemic on children’s motor skills using seven articles in 2021 [34]. The results showed that motor skills declined among children who experienced the COVID-19 pandemic because of changes in their health behavior at home, such as increased sleeping, eating, and screen time during the pandemic. Our results indicated a generally reduced proportion of children with suspected neurodevelopmental delay in the gross motor domain during the COVID-19 pandemic than before; however, these changes were only prominent in the non-Medicaid group. In the Medicaid group, the proportion of suspected neurodevelopmental delay during the COVID-19 pandemic exceeded that observed before the pandemic (Table S1). A Korean study examining time allocation after school based on SES levels revealed that children in the lowest SES group exhibited a more monotonous and imbalanced daily routine than those in the high SES group during the COVID-19 period [35]. Notable variations were noted in how the socioeconomic factors impacted the children’s developmental delay. Possibly the health behavior of the children in the low SES had been changed, such as decreasing physical activity for a long time, increasing screen time, and playing games [36–38]. A systematic review examined the relationship between SES and physical activity and sports participation among Korean children and adolescents [39]. However, further studies will be required to maintain gross motor during the pandemic in the high SES group among Koreans.

Moreover, we estimated the difference in the risk of neurodevelopmental delay based on the geographical location of residence during the COVID-19 pandemic compared to before the pandemic. The geographical location of residence is an important indicator of children’s neurodevelopment, encompassing factors such as their behaviors, access to well-childcare centers, treatment of neurodevelopmental dysfunction, and the diagnosis of neurodevelopment [17, 40]. Further, we found that the negative impacts on fine motor function, cognition, communication, social interaction, and self-care were more pronounced among urban children. Urban children may become more sedentary, socially isolated, and less stimulated than rural children who have a better chance of contact with nature. Explaining the observed differences is essential for understanding the underlying pathways and developing policies and interventions to mitigate them. This could have implications for pandemic-related measures, leading to more frequent school closures and stricter social distancing in urban areas as compared to in rural areas, given the higher transmission rates in urban settings [41, 42].

Despite the containment strategies during the pandemic, it remains essential to explore opportunities to promote language and social development in children through new experiences outside the home, including larger gatherings with family and friends or attendance at childcare centers. Additionally, this study identified a support program for healthy daily routines for children with the Medicaid group during the COVID-19 pandemic.

This study had several limitations. First, it is difficult to determine the overall developmental outcome using only K-DST results at a single time point (ages 30–36 months). Individual child development rates vary; even children with developmental delays often eventually show normal development. However, we conducted the same analysis for each K-DST visit and observed relatively higher proportions of neurodevelopmental delay during the 30–36 months compared to visits at different ages in our data (data not shown). Second, limitations of the K-DST include its nature as a screening tool rather than a diagnostic tool, relying on parents’ questionnaire responses and doctors’ assessments for overall evaluation. This introduces a degree of subjectivity in developmental evaluation. In cases where the screening result is abnormal, further detailed examination or follow-up is necessary, and caution is required in interpreting our results. Despite these limitations, the K-DST is acknowledged for its reliability and validity, highlighting its potential as an effective screening tool for neurodevelopmental disorders in infants and children in Korea [20]. Third, our dataset lacked information on the family or genetic history of neurodevelopment-related disorders and social relationships, including the presence of siblings, number of family members, parents’ occupational status, and parents’ marital status. These unmeasured residual confounders could introduce bias to the association between the COVID-19 pandemic and neurodevelopment. Fourth, obtaining precise SES estimates in population-based cohort studies is challenging, but deliberate efforts were made using diverse indicators, such as insurance type and income quintiles. The binary classification favoring the non-Medicaid group poses a limitation, potentially overlooking economic nuances in a cross-sectional approach. However, emphasizing the Medicaid group as lower SES provides insights into the association of COVID-19 with childhood neurodevelopment in the most vulnerable families. Finally, our study, elucidating socio-environmental consequences in relation to the pandemic, excluded microscopic or neurophysiological changes. Although coronavirus rarely crosses the placenta [43], coronavirus was detected in the olfactory cortex and interconnected regions with neuro-inflammation and neuronal damage [44]. Due to the lack of children’s post-mortem brain changes by virus, the underlying mechanisms of these associations remain unclear. Even without fetal viral infection, maternal inflammatory response induced by the virus might result in long-term adverse outcomes for the child and the mother. Long-term cohort studies after the pandemic may present diverse neuronal effects of viral infection of children and mothers [45].

Nevertheless, our study has several strengths. To our knowledge, this is the first study to examine the relationship between the COVID-19 pandemic and child neurodevelopment in Koreans using nationwide data. Second, the analyses further sought to adjust for potential confounders, including maternal age or maternal nationality information from a national mother-child cohort and the number of pediatric patients, which is associated with visits for screened neurodevelopment tests.

## Conclusions

In conclusion, this study found an association between the experience of COVID-19 and a higher risk of neurodevelopmental delay in the communication and social interaction domains among children aged 30–36 months. Notably, children with Medicaid group were associated with a higher risk of neurodevelopmental delay than those with non-Medicaid group. These findings underscore the need for concern regarding the neurodevelopment of children who experienced the COVID-19 pandemic and highlight the necessity for increased training in specific domains among Medicaid children, which requires the joint efforts of both parents and child healthcare practitioners.

### Electronic supplementary material

Below is the link to the electronic supplementary material.

Supplementary Material 1

Supplementary Material 2

## Data Availability

The data that support the findings of this study are available from the National Health Insurance Service of the Republic of Korea, but restrictions apply to the availability of these data, which were used under license for the current study and thus are not publicly available.
