# Supplementary material for: Association between the COVID-19 pandemic and childhood development aged 30 to 36 months in South Korea, based on the National health screening program for infants and children database
Source: BMC Public Health. 2024 Apr 9;24:989. doi: 10.1186/s12889-024-18361-9 (PMC11003091; doi:10.1186/s12889-024-18361-9)
Supplement: Supplementary file 1 — Supplementary Material 1 [file 12889_2024_18361_MOESM1_ESM.pdf]

(30~32months)

K-DST

Korean Developmental Screening Test for Infants & Children

This screening Test is part of the medical research of infants and children of the ministry of health and welfare and Korea Centers for Disease and Control and Prevention and was developed by experts under the auspices of Korean National Institute for Pediatrics and Korean Society of Pediatric Rehabilitation and Developmental Medicine, Psychologists etc.

Revised Version

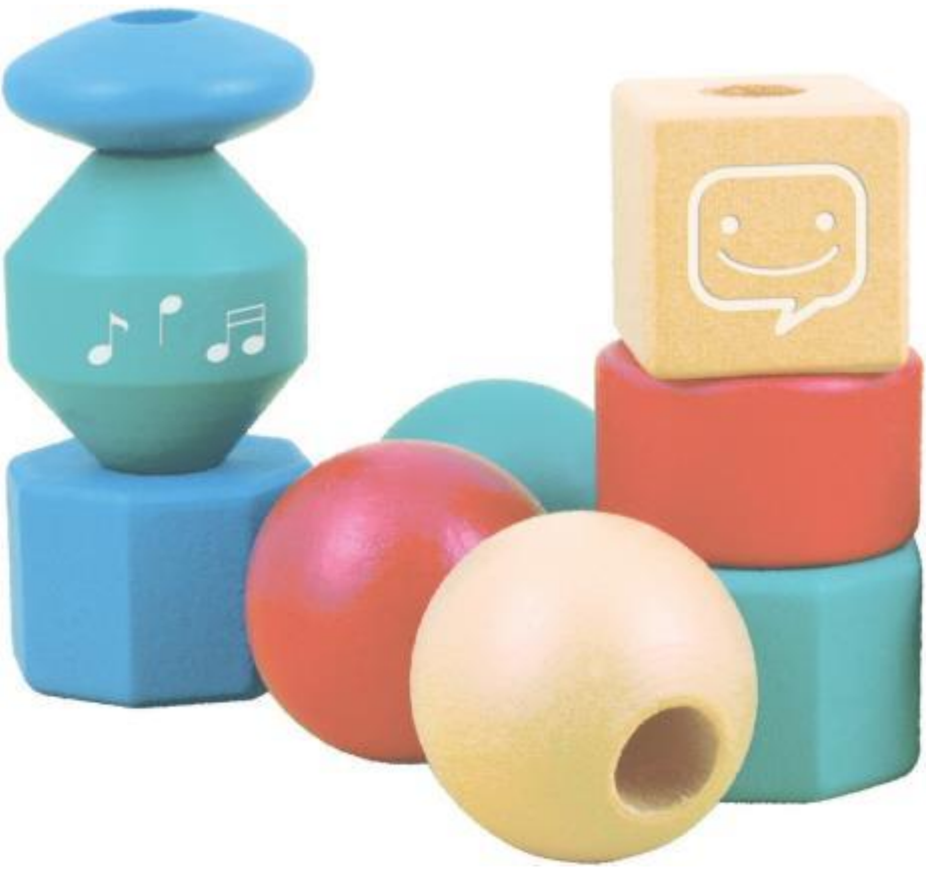

This evaluation and its publication rights belong to the Ministry of Health and Welfare and Korea Centers for Disease Control and Prevention may not be changed copied, sold, used, added or otherwise commercialized without primion's prior written approval. Except medical treatment, at infant toddler care centers, medical child care, infants and toddlers medical care, rehabilitative medical care and developmental screening tests for infants and children.

Korean Developmental Screening Test for Infants and Children (30~32months)

✣ Enter the respective situation. Mark the empty fields ☑.

Name of child		(m, f)	Interviewee	□ Mother □ Father □ Grandmother □ Grandfather □ Others( )
Date of birth	year month		day (prebirth, date of birth:	year month day)
Parent's information (optional)	Mother	age: (years)	Degree:	□ Postgraduate □ Graduate □ Drop out<br>□ High school graduate □ Junior high graduate
	Father	age: (years)	Degree:	□ Postgraduate □ Graduate □ Drop out<br>□ High school graduate □ Junior high graduate
Is the child is physically or developmentally challenged?			□ No	□ Yes (what sort of developmentally challenge?)

** This questionnaire is related to 30-32 month old infants. If the age of the child does not coincide, you have to replace the questionnaire.

✣ Choose one of the four answers below.

If you don't know the answer, you can check at the infant and then answer the question.

Can do well ③	Can do ②	Can do not well ①	Absolutely can't do ⓪

The following questions are about "what the infant can do".
If the infant is able to do a certain act but has not done it well due to other reasons, check "Can do".

e.g., The infant seems to have an ability to use scissors, but you have never let him/her use them before. The infant seems to be able to build with blocks, but you don't have such toys (blocks) in your house or he/she doesn't like playing with them.

GROSS MOTOR SKILLS

1	Jumps from bottom step	③ ② ① ⓪
2	Throws tennis ball overhead while standing 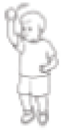	③ ② ① ⓪
3	Lifts his/her heels and walks more than four steps on his/her tiptoes	③ ② ① ⓪
4	The infant climbs stairs one step at a time by putting both feet on the same stair without holding onto the railing. (Check "Can do well" or "Can do" if the infant climbs stairs step by step alternately (one foot at a time).) 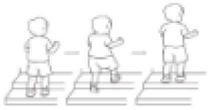	③ ② ① ⓪
5	Imitates one foot standing 1 second 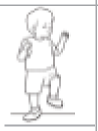	③ ② ① ⓪
6	The infant goes down stairs one step at a time by putting both feet on the same stair without holding onto anything. (Check "Can do well" or "Can do" if the infant goes down stairs step by step alternately (one foot at a time).) 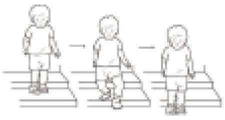	③ ② ① ⓪
7	Climbs stairs step by step alternately without holding anything 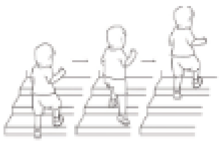	③ ② ① ⓪
8	Catches ball with both hands 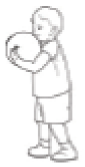	③ ② ① ⓪

Can do well ③	Can do ②	Can do not well ①	Absolutely can't do ⓪

FINE MOTOR SKILLS

1	Opens door by turning mob	③ ② ① ⓪
2	Holds the lower part of a (colored) pencil	③ ② ① ⓪
3	Cuts a across a piece of paper, graps scissor and paper in each hand	③ ② ① ⓪
4	Strings lace in shoelace hole or perls on a cordon, puts it off. 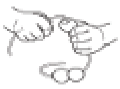	③ ② ① ⓪
5	Imitates vertical strokes (except in cases where the infant draws by tracing over lines already drawn) 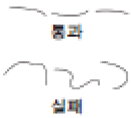	③ ② ① ⓪
6	Holds a (colored) pencil, crayon, or pen using his/her thumb and another finger	③ ② ① ⓪
7	Undresses itself and a doll upon request.	③ ② ① ⓪
8	Imitates a circle (without showing)	③ ② ① ⓪

COGNITION

1	Matches the correct color of coloured blocks (red, yellow, blue)	③ ② ① ⓪
2	Knows the measurement “much-less”"(e.g.: knows 6 candies are more than 2.) ‘	③ ② ① ⓪
3	Matches a 6-piece puzzle.	③ ② ① ⓪
4	Differentiates the long stroke from the short stroke.	③ ② ① ⓪
5	Understands "2" (e.g. gives 2 of 3 candies on the table).	③ ② ① ⓪
6	Differentiates different sized objects, ‘biggest’, ‘medium size’, ‘smallest’.	③ ② ① ⓪
7	Understands ‘inside, outside, between’ (put cup into the container).	③ ② ① ⓪
8	Remembers two different requests and carries out (e.g. “Throw away the paper towel, get me a book”).	③ ② ① ⓪

LANGUAGE

1	Follows request "put it on the table" without showing.	③ ② ① ⓪
2	Among ‘inside’, ‘above’, ‘below’, ‘backwards’ understands at least two	③ ② ① ⓪
3	Reading a book expresses retells the plot. (e.g.:Mother: "What is the dog doing?" Child: "Sleeping", "Eating", "Crying" etc.	③ ② ① ⓪
4	Being asked "What 's your name?" say its full name(forename/surename).	③ ② ① ⓪
5	Uses the past tense: have done.	③ ② ① ⓪
6	Has a mini conversations.	③ ② ① ⓪
7	Knows what ‘pretty’ and ‘afraid’ means.	③ ② ① ⓪
8	Knows the appellations ‘grandpa’, ‘grandma’, ‘big brother’ etc.	③ ② ① ⓪

✣ Choose one of the four answers below.

If you don't know the answer, you can check at the infant and then answer the question.

Can do well ③	Can do ②	Can do not well ①	Absolutely can't do ⓪

SOCIABILITY

1	Says "sorry" and "thank you" upon request	③ ② ① ⓪	5	Imitates adults group behaviour. (e.g. Play the train game, catch-the-tail game, ladder and tunnel game, etc.)	③ ② ① ⓪
2	Imitates other children's play	③ ② ① ⓪	6	Waits for its turn(playground, slide)	③ ② ① ⓪
3	Says “I feel good” or “I feel bad” when he/she feels so	③ ② ① ⓪	7	Comforts other children in distress	③ ② ① ⓪
4	Plays games in group of 3-4("chase" and "hide and seek")	③ ② ① ⓪	8	Plays with friends with the flow of a story (e.g. “Playing with dolls”, “playing school”, etc.)	③ ② ① ⓪

RESPONSIBLITY

1	Uses napkin after eating and doesn't use its sleeves.	③ ② ① ⓪	5	Soaps/washes hands alone when water is turned on	③ ② ① ⓪
2	If you put the feet of the infant into their pants a little bit, he/she pulls them up to the waist.	③ ② ① ⓪	6	Puts on socks	③ ② ① ⓪
3	Remains dry during the day	③ ② ① ⓪	7	Feeds itself	③ ② ① ⓪
4	Holds back stool during the day	③ ② ① ⓪	8	Dresses itself (T-shirt)	③ ② ① ⓪

FURTHER QUESTION

Yes 1	No 0

1	Can’t walk.	1	0	4	The infant does not look at you even though you call his/her name. (except in cases where the infant has hearing impairments or does not look at you because he/she is focusing on something else)	1	0
2	Words make no sense. (e.g. “Bow-wow”, “momma”, “water”, etc.)	1	0	5	The infant does not do any behaviors to attract adults’ attention (e.g., Pointing his/her finger at an item and seeing his/her guardian’s reactions, bringing and showing an item, pestering someone to play with him/her, calling someone by making sounds, etc.)	1	0
3	The infant does not make eye contact well with his/her guardians. (except in cases where the infant does not make eye contact because he/she is focusing on something else)	1	0				

Evaluation Chart (30~32 months)

Name of child		(m, f)	Date of preparation	year month day
Date of birth	year month day (prebirth, date of birth: year month day)			
Interviewee		□Mother □Father □Grandmother □ Grandfather □Others( )		

Summary report

Category / Domain	1	2	3	4	5	6	7	8	Total score	Transfer points		
										A	B	C
Gross motor skills										15	19	24
Fine motor skills										12	17	23
Cognition										10	16	24
Language										9	18	24
Sociability										11	17	24
Responsibility										10	15	23

Further question

1 = Yes 0 = No

Question	1 (M)	2 (M)	3 (S)	4 (S)	5 (S)
Result	1<br>0	1<br>0	1<br>0	1<br>0	1<br>0

Evaluation result

Scoring

1. The Scoring is based on four steps.
   (can do well = 3points can do = 2points, can't do well = 1point absolutely can't do = 0 points)
2. Each domain is united with the points of difficulty of the question and recorded.
3. The evaluation of the total score is based on the transfer points of each domain is scored on four steps.
   ① advanced evaluation recommended ② monitoring is required ③ peer level ④ fast level

Date

preparing person: signa

t

u

r

e

(33~35months)

K-DST

Korean Developmental Screening Test for Infants & Children

This screening Test is part of the medical research of infants and children of the ministry of health and welfare and Korea Centers for Disease and Control and Prevention and was developed by experts under the auspices of Korean National Institute for Pediatrics and Korean Society of Pediatric Rehabilitation and Developmental Medicine, Psychologists etc.

Revised Version

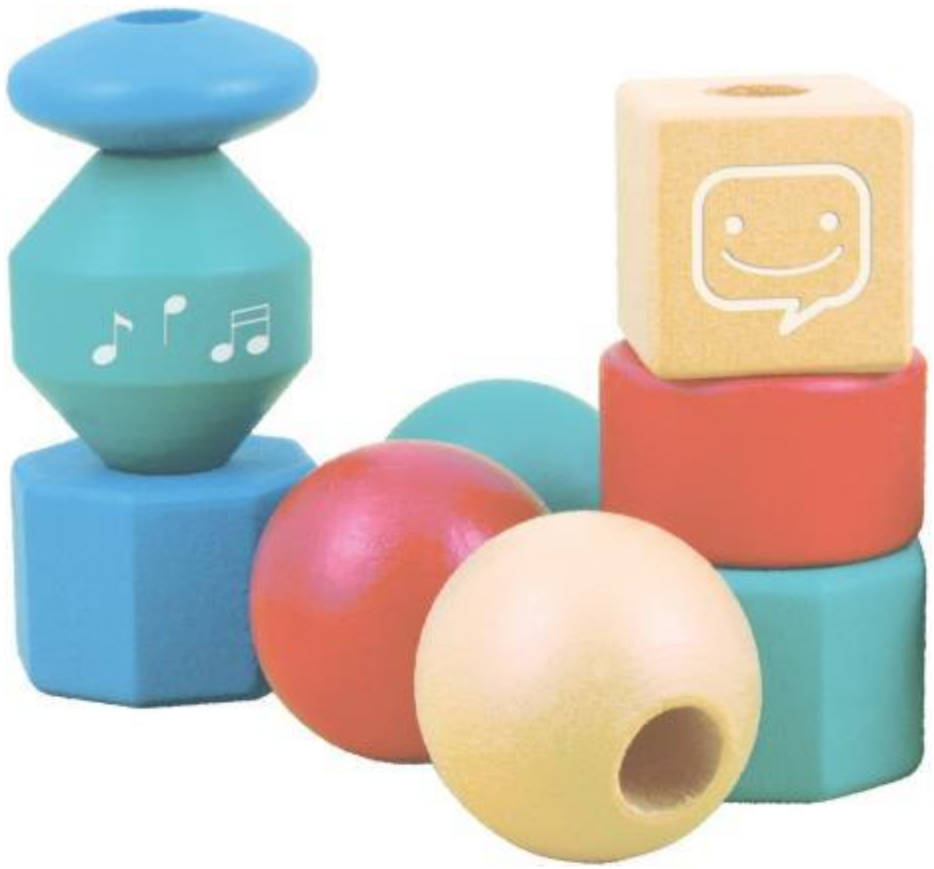

This evaluation and its publication rights belong to the Ministry of Health and Welfare and Korea Centers for Disease Control and Prevention may not be changed copied, sold, used, added or otherwise commercialized without primion's prior written approval. Except medical treatment, at infant toddler care centers, medical child care, infants and toddlers medical care, rehabilitative medical care and developmental screening tests for infants and children.

Korean Developmental Screening Test for Infants and Children (33~35months)

✣ Enter the respective situation. Mark the empty fields ☑.

Name of child		(m, f)	Interviewee	□ Mother □ Father □ Grandmother □ Grandfather □ Others( )
Date of birth	year month day (prebirth, date of birth: year month day)			
Parent's information (optional)	Mother	age: (years)	Degree: □ Postgraduate □ Graduate □ Drop out □ High school graduate □ Junior high graduate	
	Father	age: (years)	Degree: □ Postgraduate □ Graduate □ Drop out □ High school graduate □ Junior high graduate	
Is the child is physically or developmentally challenged?	□ No □ Yes (what sort of developmentally challenge?)			

** This questionnaire is related to 33-35 month old infants. If the age of the child does not coincide, you have to replace the questionnaire.

✣ Choose one of the four answers below.

If you don't know the answer, you can check at the infant and then answer the question.

Can do well ③	Can do ②	Can do not well ①	Absolutely can't do ⓪

The following questions are about "what the infant can do".
If the infant is able to do a certain act but has not done it well due to other reasons, check "Can do".
e.g., The infant seems to have an ability to use scissors, but you have never let him/her use them before. The infant seems to be able to build with blocks, but you don't have such toys (blocks) in your house or he/she doesn't like playing with them.

GROSS MOTOR SKILLS

1	Imitates one foot standing 1 second 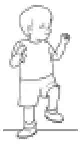	③ ② ① ⓪
2	The infant goes down stairs one step at a time by putting both feet on the same stair without holding onto anything. (Check "Can do well" or "Can do" if the infant goes down stairs step by step alternately (one foot at a time).) 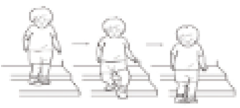	③ ② ① ⓪
3	Climbs stairs step by step alternately without holding anything 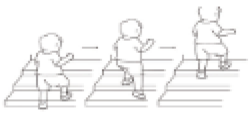	③ ② ① ⓪
4	Catches a big ball 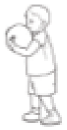	③ ② ① ⓪
5	Peddles tricycle short distances	③ ② ① ⓪
6	Goes straight a line.	③ ② ① ⓪
7	Jumps forward with 2-footed take-off and landing 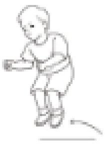	③ ② ① ⓪
8	Imitates one foot standing 3 seconds 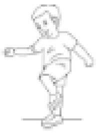	③ ② ① ⓪

Can do well ③	Can do ②	Can do not well ①	Absolutely can't do ⓪

FINE MOTOR SKILLS

1	Strings lace in shoelace hole or perls on a cordon, puts it off. 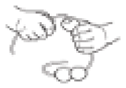	③ ② ① ⓪
2	Imitates vertical strokes (except in cases where the infant draws by tracing over lines already drawn) 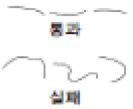	③ ② ① ⓪
3	Undresses itself and a doll upon request.	③ ② ① ⓪
4	Imitates a circle without showing	③ ② ① ⓪

5	Imitates a cross without showing, only strokes 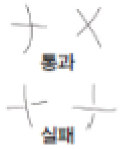	③ ② ① ⓪
6	Folds a piece of paper two times(can be uneven).	③ ② ① ⓪
7	Draws a stroke	③ ② ① ⓪
8	If you show the infant a square, he/she draws one without any demonstration. (It is okay even if the lines intersect with each other. However, it does not count if the angles are round or narrow.) 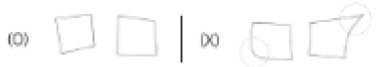	③ ② ① ⓪

COGNITION

1	Matches a 6-piece puzzle.	③ ② ① ⓪
2	Differentiates different sized objects, 'biggest', 'medium size', 'smallest'.	③ ② ① ⓪
3	Understands 'inside, outside, between' (put cup into the container).	③ ② ① ⓪
4	Remembers two different requests and carries out (e.g. "Throw away the paper towel, get me a book").	③ ② ① ⓪

5	Knows its gender.	③ ② ① ⓪
6	Understands '3' (e.g. gives 3 of several candies on the table).	③ ② ① ⓪
7	Matches mixed category pictures as fruits, vehicles, furnitures,	③ ② ① ⓪
8	Knows the meaning of 'most' and 'less.'	③ ② ① ⓪

LANGUAGE

1	Reading a book it can express what is happening. (e.g.: Mother:"What is the dog doing?" Child: "Sleeping","Eating","Crying" etc. retells the situation.	③ ② ① ⓪
2	If it is being asked "What 's your name?" say its full name(forename/surename).	③ ② ① ⓪
3	Can use four word sentences (e.g. want go toy store).	③ ② ① ⓪

4	Uses the past tense: have done.	③ ② ① ⓪
5	Can have a mini conversation.	③ ② ① ⓪
6	Knows what 'pretty' and 'afraid' means.	③ ② ① ⓪
7	Knows the appellation 'grandpa', 'grandma', 'big brother', 'big sister', little sister.	③ ② ① ⓪
8	Can name three words of the same category (e.g. Been asked for animals, answers 'dog', 'cat', 'elephant').	③ ② ① ⓪

✢ Choose one of the four answers below.
If you don't know the answer, you can check at the infant and then answer the question.

Can do well ③	Can do ②	Can do not well ①	Absolutely can't do ⓪

SOCIABILITY

1	Imitates other children's play	③ ② ① ⓪
2	Plays games in group of 3-4("chase" and "hide and seek")	③ ② ① ⓪
3	Imitates adults group behaviour. (e.g. Play the train game, catch-the-tail game, ladder and tunnel game, etc.)	③ ② ① ⓪
4	Waits for its turn (playground, slide)	③ ② ① ⓪
5	Comforts other children in distress	③ ② ① ⓪
6	Plays with friends with the flow of a story (e.g. "Playing with dolls", "playing school", etc.)	③ ② ① ⓪
7	Knows at least two names of his/her friends	③ ② ① ⓪
8	Can explain a simple game.	③ ② ① ⓪

RESPONSIBILITY

1	Holds back stool during the day	③ ② ① ⓪
2	Soaps/washes hands alone when water is turned on	③ ② ① ⓪
3	Buttons front-opening clothing	③ ② ① ⓪
4	Puts on socks	③ ② ① ⓪
5	Feeds itself	③ ② ① ⓪
6	Dresses itself(T-shirt)	③ ② ① ⓪
7	Puts rubber boots on	③ ② ① ⓪
8	Differentiates back und front of its clothes.	③ ② ① ⓪

FURTHER QUESTION

		Yes 1	No 0
1	Used words make no sense. (e.g. "Bow-wow", "momma", "water", etc.)	1	0
2	The infant does not make eye contact well with his/her guardians. (except in cases where the infant does not make eye contact because he/she is focusing on something else)	1	0
3	The infant does not look at you even though you call his/her name. (except in cases where the infant has hearing impairments or does not look at you because he/she is focusing on something else)	1	0
4	The infant does not do any behaviors to attract adults' attention (e.g., Pointing his/her finger at an item and seeing his/her guardian's reactions, bringing and showing an item, pestering someone to play with him/her, calling someone by making sounds, etc.)	1	0

Evaluation Chart (33~35 months)

Name of child		(m, f)	Date of preparation	year month day
Date of birth	year month day (prebirth, date of birth: year month day)			
Interviewee		□Mother □Father □Grandmother □ Grandfather □Others( )		

Summary report

Category / Domain	1	2	3	4	5	6	7	8	Total score	Transfer points		
										A	B	C
Gross motor skills										14	18	24
Fine motor skills										10	15	23
Cognition										11	17	24
Language										9	19	24
Sociability										11	17	24
Responsibility										12	17	24

Further question

[illegible]

Question	1 (L)	2 (S)	3 (S)	4 (S)
Result	1<br>0	1<br>0	1<br>0	1<br>0

Evaluation result

Scoring

1. The Scoring is based on four steps.
   (can do well = 3points can do = 2points, can't do well = 1point absolutely can't do = 0 points)
2. Each domain is united with the points of difficulty of the question and recorded.
3. The evaluation of the total score is based on the transfer points of each domain is scored on four steps.
   ① advanced evaluation recommended ② monitoring is required ③ peer level ④ fast level

Date

t

u

r

e

(36~41months)

K-DST

Korean Developmental Screening Test for Infants & Children

This screening Test is part of the medical research of infants and children of the ministry of health and welfare and Korea Centers for Disease and Control and Prevention and was developed by experts under the auspices of Korean National Institute for Pediatrics and Korean Society of Pediatric Rehabilitation and Developmental Medicine, Psychologists etc.

Revised Version

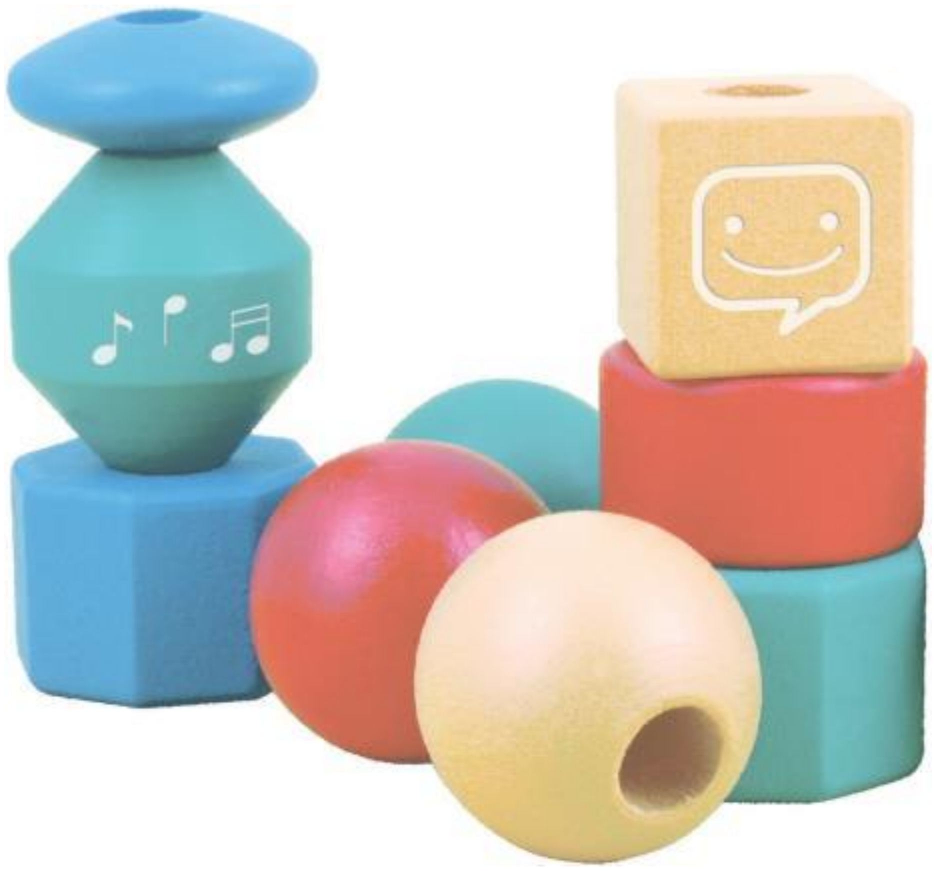

This evaluation and its publication rights belong to the Ministry of Health and Welfare and Korea Centers for Disease Control and Prevention may not be changed copied, sold, used, added or otherwise commercialized without primion's prior written approval. Except medical treatment, at infant toddler care centers, medical child care, infants and toddlers medical care, rehabilitative medical care and developmental screening tests for infants and children.

Korean Developmental Screening Test for Infants and Children (36~41months)

✢ Enter the respective situation. Mark the empty fields ☑.

Name of child	(m, f)	Interviewee	□ Mother □ Father □ Grandmother □ Grandfather □ Others( )	
Date of birth	year month	day (prebirth, date of birth:	year month day)	
Parent's information (optional)	Mother	age: (years)	Degree: □ Postgraduate □ Graduate □ Drop out □ High school graduate □ Junior high graduate	
	Father	age: (years)	Degree: □ Postgraduate □ Graduate □ Drop out □ High school graduate □ Junior high graduate	
Is the child is physically or developmentally challenged?	□ No	□ Yes (what sort of developmentally challenge?)		

** This questionnaire is related to 36-41 month old infants. If the age of the child does not coincide, you have to replace the questionnaire.

✢ Choose one of the four answers below.

If you don't know the answer, you can check at the infant and then answer the question.

Can do well ③	Can do ②	Can do not well ①	Absolutely can't do ⓪

The following questions are about "what the infant can do".
If the infant is able to do a certain act but has not done it well due to other reasons, check "Can do".
e.g., The infant seems to have an ability to use scissors, but you have never let him/her use them before. The infant seems to be able to build with blocks, but you don't have such toys (blocks) in your house or he/she doesn't like playing with them.

GROSS MOTOR SKILLS

1	Climbs stairs step by step alternately without holding anything 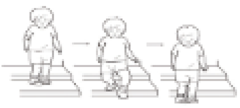	③ ② ① ⓪
2	Catches a big ball 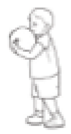	③ ② ① ⓪
3	Peddles tricycle short distances	③ ② ① ⓪
4	Goes straight a line.	③ ② ① ⓪
5	Jumps forward with 2-footed take-off and landing 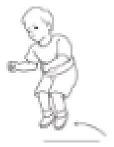	③ ② ① ⓪
6	Imitates one foot standing 3 seconds 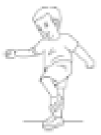	③ ② ① ⓪
7	Moves on "ride-on" toy without pedals	③ ② ① ⓪
8	Hops on one foot forward 2-3 times	③ ② ① ⓪

Can do well ③	Can do ②	Can do not well ①	Absolutely can't do ⓪

FINE MOTOR SKILLS

1	Undresses itself and a doll upon request	③ ② ① ⓪
2	Imitates a circle (without showing)	③ ② ① ⓪
3	Folds a piece of paper two times (can be uneven).	③ ② ① ⓪
4	Can draw the rest of a line.	③ ② ① ⓪
5	If you show the infant a square, he/she draws one without any demonstration. (It is okay even if the lines intersect with each other. However, it does not count if the angles are round or narrow.) 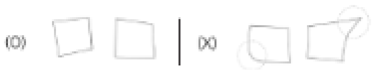	③ ② ① ⓪
6	Can cut along the line with scissors.	③ ② ① ⓪
7	Can untwist a cap.	③ ② ① ⓪
8	Imitates a cross without showing, only strokes 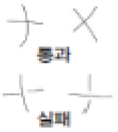	③ ② ① ⓪

COGNITION

1	Remembers two different requests and carries out (e.g. "Throw away the paper towel, get me a book").	③ ② ① ⓪
2	Knows its gender.	③ ② ① ⓪
3	Understands '3' (e.g. gives 3 of several candies on the table).	③ ② ① ⓪
4	Matches mixed category pictures as fruits, vehicles, furnitures,	③ ② ① ⓪
5	Knows the meaning of 'most' and 'less.'	③ ② ① ⓪
6	Can deliver speech	③ ② ① ⓪
7	Differentiates at least five different colours.	③ ② ① ⓪
8	Draws a person (e.g. Mother, father consists of three parts of the body. It takes about three minutes. 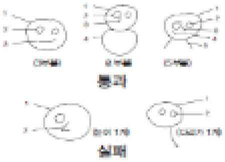	③ ② ① ⓪

LANGUAGE

1	If it is being asked "What 's your name?" say its full name(forename/surename).	③ ② ① ⓪
2	Can use four word sentences (e.g. want go buy at toy store).	③ ② ① ⓪
3	Uses the past tense: have done.	③ ② ① ⓪
4	Can have a mini conversation.	③ ② ① ⓪
5	Can build a proper sentence (e.g.: "The dog ate the cookie")	③ ② ① ⓪
6	Can use the particles 'un, nun, lee, ga' properly and build a correct sentence (e.g.: "The cat says 'meow'", "I like my friend").	③ ② ① ⓪
7	Can name three words of the same category (e.g. Been asked for animals, answers 'dog', 'cat', 'elephant').	③ ② ① ⓪
8	Can use the phrases 'will do' and 'want to do' correctly in the future sense.	③ ② ① ⓪

✣ Choose one of the four answers below.

If you don't know the answer, you can check at the infant and then answer the question.

Can do well ③	Can do ②	Can do not well ①	Absolutely can't do ⓪

SOCIABILITY

1	Imitates adults group behaviour. (e.g. Play the train game, catch-the-tail game, ladder and tunnel game, etc.)	③ ② ① ⓪
2	Waits for its turn (playground, slide)	③ ② ① ⓪
3	Comforts other children in distress	③ ② ① ⓪
4	Plays with friends with the flow of a story (e.g. "Playing with dolls", "playing school", etc.)	③ ② ① ⓪
5	Can explain a simple game.	③ ② ① ⓪
6	Waits for its turn and shares with playmates	③ ② ① ⓪
7	Can report other children's misbehaviour (e.g. "____ brought some snacks.")	③ ② ① ⓪
8	Can play various situation games as 'household', 'shop', 'school', 'hospital'.	③ ② ① ⓪

RESPONSIBILITY

1	Puts socks on	③ ② ① ⓪
2	Feeds itself	③ ② ① ⓪
3	Dresses itself(unbuttoned T-shirt)	③ ② ① ⓪
4	Puts rubber boots on.	③ ② ① ⓪
5	Differentiates back und front of its clothes.	③ ② ① ⓪
6	Dresses itself(T-shirt)	③ ② ① ⓪
7	Buttons front-opening clothing	③ ② ① ⓪
8	Can wash its hand and dry with a towel.	③ ② ① ⓪

FURTHER QUESTION

		Yes 1	No 0
1	Used words make no sense. (e.g. "Bow-wow", "momma", "water", etc.)	1	0
2	Can't make two word sentences. (e.g. "Give me something to eat", "Let's drink some milk", etc.)	1	0
3	The infant does not make eye contact well with his/her guardians. (except in cases where the infant does not make eye contact because he/she is focusing on something else)	1	0
4	The infant does not look at you even though you call his/her name. (except in cases where the infant has hearing impairments or does not look at you because he/she is focusing on something else)	1	0
5	The infant does not do any behaviors to attract adults' attention (e.g., Pointing his/her finger at an item and seeing his/her guardian's reactions, bringing and showing an item, pestering someone to play with him/her, calling someone by making sounds, etc.)	1	0
6	Is not interested in peers, even it peers are in its surrounding, the child doesn't observe and doesn't try to imitate them.	1	0
7	The infant does not do role playing consisting of two or more roles. (e.g. Doctor and patient, mom and dad, etc.)	1	0

Evaluation Chart (36~41 months)

Name of child		(m, f)	Date of preparation	year month day
Date of birth	year month day (prebirth, date of birth: year month day)			
Interviewee	□Mother □Father □Grandmother □ Grandfather □Others( )			

Evaluation result

Category / Domain	1	2	3	4	5	6	7	8	Total score	Transfer point		
										A	B	C
Gross motor skills										12	16	23
Fine motor skills										10	15	23
Cognition										10	16	23
Language										7	19	24
Sociability										12	17	24
Responsibility										11	15	23

Further question

1= Yes 0=No

Question	1 (L)	2 (L)	3 (S)	4 (S)	5 (S)	6 (S)	7 (S)
Result	1 0	1 0	1 0	1 0	1 0	1 0	1 0

Evaluation result

Scoring

1. The Scoring is based on four steps.
   (can do well = 3points can do = 2points, can't do well = 1point absolutely can't do = 0 points)
2. Each domain is united with the points of difficulty of the question and recorded.
3. The evaluation of the total score is based on the transfer points of each domain is scored on four steps.
   ① advanced evaluation recommended ② monitoring is required ③ peer level ④ fast level

Date of preparation: ________________

Preparing person:__________________signature
