# Supplementary material for: Association between the COVID-19 pandemic and childhood development aged 30 to 36 months in South Korea, based on the National health screening program for infants and children database
Source: BMC Public Health. 2024 Apr 9;24:989. doi: 10.1186/s12889-024-18361-9 (PMC11003091; doi:10.1186/s12889-024-18361-9)
Supplement: Supplementary file 2 — Supplementary Material 2 [file 12889_2024_18361_MOESM2_ESM.docx]

Table S1. The proportion of neurodevelopmental delay between before and during COVID-19 period according to SES level among 30-36 months old.

|  | Medicaid group | | | Non-Medicaid group | | |
| --- | --- | --- | --- | --- | --- | --- |
| The 7 domains of the K-DST | Before  COVID-19 pandemic | During COVID-19 pandemic | P-value | Before  COVID-19 pandemic | During COVID-19 pandemic | P-value |
|  | N(%) | N(%) |  | N(%) | N(%) |  |
| Any | 1,546(25.8) | 1,523(30.82) | <0.0001 | 90,499(16.09) | 86,444(17.04) | <0.0001 |
| Gross Motor | 560(9.34) | 519(10.50) | 0.043 | 23,545(4.19) | 20,321(4.00) | <0.0001 |
| Fine Motor | 755(12.60) | 770(15.58) | <0.0001 | 38,218(6.79) | 36,061(7.11) | <0.0001 |
| Cognition | 840(14.02) | 815(16.49) | 0.0003 | 32,726(5.82) | 31,047(6.12) | <0.0001 |
| Communication | 1,091(18.20) | 1,149(23.25) | <0.0001 | 48,285(8.58) | 50,121(9.88) | <0.0001 |
| Social Interaction | 828(13.82) | 841(17.02) | <0.0001 | 37,492(6.67) | 38,188(7.53) | <0.0001 |
| Self-Care | 660(11.01) | 651(13.17) | 0.001 | 40,054(7.12) | 38,482(7.58) | <0.0001 |

Note: Non-Medicaid group was indicated Self-employed insured or insured employee.

Table S2. The proportion of neurodevelopmental delay between before and during COVID-19 period according to residence level among 30-36 months old.

|  | Urban | | | Rural | | |
| --- | --- | --- | --- | --- | --- | --- |
| The 7 domains of the K-DST | Before  COVID-19 pandemic | During COVID-19 pandemic | P-value | Before  COVID-19 pandemic | During COVID-19 pandemic | P-value |
|  | N(%) | N(%) |  | N(%) | N(%) |  |
| Any | 39,067(16.22) | 37,415(17.67) | <.0001 | 52,978(16.17) | 50,552(16.81) | <.0001 |
| Gross Motor | 10,155(4.22) | 8,935(4.22) | 0.943 | 13,950(4.26) | 11,905(3.96) | <.0001 |
| Fine Motor | 16,619(6.9) | 15,821(7.47) | <.0001 | 22,354(6.82) | 21,010(6.99) | 0.010 |
| Cognition | 13,604(5.65) | 12,946(6.12) | <.0001 | 19,962(6.09) | 18,916(6.29) | 0.001 |
| Communication | 20,369(8.46) | 20,996(9.92) | <.0001 | 29,007(8.85) | 30,274(10.07) | <.0001 |
| Social Interaction | 16,365(6.79) | 16,539(7.81) | <.0001 | 21,955(6.7) | 22,490(7.48) | <.0001 |
| Self-Care | 17,736(7.36) | 17,102(8.08) | <.0001 | 22,978(7.01) | 22,031(7.33) | <.0001 |

Table S3. Multivariable logistic regression models for screening for neurodevelopmental delay between before and during COVID-19 period **according to SES level** among 30-36 months old.

|  | Screened for neurodevelopmental delay | | | | | | | | Interaction term | |
| --- | --- | --- | --- | --- | --- | --- | --- | --- | --- | --- |
| The 7 domains of the K-DST | Medicaid group | | | | Non-Medicaid group | | | |  |  |
|  | OR (95% CI) | P-value | aOR (95% CI) | P-value | OR (95% CI) | P-value | aOR (95% CI) | P-value | Wald Chi-Square | p-value |
| Any domain | 1.28(1.18, 1.39) | <0.0001 | 1.31(1.20, 1.43) | <0.0001* | 1.07(1.06, 1.08) | <0.0001 | 1.11(1.10, 1.12) | <0.0001* | 14.00 | 0.0002 |
| Gross Motor | 1.14(1.00, 1.29) | 0.059 | 1.11(0.97, 1.26) | 0.044 | 0.96(0.94, 0.97) | <0.0001 | 0.95(0.93, 0.97) | <0.0001* | 4.32 | 0.038 |
| Fine Motor | 1.28(1.15, 1.43) | <0.0001 | 1.28(1.15, 1.44) | <0.0001* | 1.05(1.03, 1.07) | <0.0001 | 1.07(1.06, 1.09) | <0.0001* | 9.03 | 0.003 |
| Cognition | 1.21(1.09, 1.35) | 0.0004 | 1.23(1.10, 1.37) | 0.0003* | 1.06(1.04, 1.07) | <0.0001 | 1.09(1.08, 1.11) | <0.0001* | 4.07 | 0.044 |
| Communication | 1.36(1.24, 1.49) | <0.0001 | 1.41(1.28, 1.55) | <0.0001* | 1.17(1.15, 1.18) | <0.0001 | 1.20(1.19, 1.22) | <0.0001* | 8.38 | 0.004 |
| Social Interaction | 1.28(1.15, 1.42) | <0.0001 | 1.30(1.16, 1.45) | <0.0001* | 1.14(1.12, 1.16) | <0.0001 | 1.15(1.13, 1.17) | <0.0001* | 3.06 | 0.080 |
| Self-Care | 1.23(1.09, 1.38) | 0.001 | 1.29(1.14, 1.45) | 0.001* | 1.07(1.06, 1.09) | <0.0001 | 1.14(1.12, 1.15) | <0.0001* | 3.44 | 0.064 |

Adjusting for children’s sex, age(continuous), residence level, preterm birth or LBW, maternal age group, mother’s nationality, number of pediatricians per population of children, delayed visit, and section type of survey

* Significant after Bonferroni correction for 16 risk factors examined (p-value < 0.003).

Table S4. Multivariable logistic regression models for screening for neurodevelopmental delay between before and during COVID-19 period among children aged 30–36 months according to the SES level (combined by annual income and insurance type).

| The 7 domains of the K-DST | SES level=1 | | SES level=2 | | SES level=3 | | SES level=4 | | SES level=5 | |
| --- | --- | --- | --- | --- | --- | --- | --- | --- | --- | --- |
|  | aOR | P-value | aOR | P-value | aOR | P-value | aOR | P-value | aOR | P-value |
| Any domain | 1.31(1.20, 1.43) | <0.0001* | 1.14(1.10, 1.19) | <0.0001* | 1.13(1.09, 1.16) | <0.0001* | 1.12(1.10, 1.15) | <0.0001* | 1.09(1.08, 1.11) | <0.0001* |
| Gross Motor | 1.10(0.97, 1.26) | 0.142 | 1.02(0.95, 1.09) | 0.553 | 1.00(0.94, 1.06) | 0.952 | 0.95(0.91, 0.99) | 0.015 | 0.93(0.90, 0.95) | <0.0001* |
| Fine Motor | 1.28(1.15, 1.44) | <0.0001* | 1.09(1.03, 1.15) | 0.003 | 1.10(1.06, 1.16) | <0.0001* | 1.07(1.04, 1.11) | <0.0001* | 1.05(1.03, 1.07) | <0.0001* |
| Cognition | 1.23(1.10, 1.37) | 0.0003* | 1.13(1.07, 1.19) | <0.0001* | 1.12(1.07, 1.17) | <0.0001* | 1.10(1.06, 1.14) | <0.0001* | 1.07(1.05, 1.10) | <0.0001* |
| Communication | 1.41(1.28, 1.55) | <0.0001* | 1.23(1.17, 1.29) | <0.0001* | 1.21(1.16, 1.25) | <0.0001* | 1.22(1.18, 1.26) | <0.0001* | 1.18(1.16, 1.21) | <0.0001* |
| Social Interaction | 1.30(1.16, 1.45) | <0.0001* | 1.15(1.09, 1.22) | <0.0001* | 1.21(1.16, 1.27) | <0.0001* | 1.14(1.10, 1.18) | <0.0001* | 1.13(1.10, 1.15) | <0.0001* |
| Self-Care | 1.29(1.14, 1.45) | <0.0001* | 1.14(1.08, 1.21) | <0.0001* | 1.18(1.13, 1.23) | <0.0001* | 1.14(1.10, 1.18) | <0.0001* | 1.12(1.10, 1.14) | <0.0001* |

Adjusting for children’s sex, age(continuous), residence level, preterm birth or LBW, maternal age group, mother’s nationality, number of pediatricians per population of children, delayed visit, and section type of survey

Note: SES level=1; Medicaid, SES level=2; self-employed & 1^st^-10^th^ of annual income, SES level=3; self-employed & 11^th^-20^th^ of annual income, SES level=4; insured employee & 1^st^-10^th^ of annual income, SES level=5; insured employee & 11^th^-20^th^ of annual income)

* Significant after Bonferroni correction for 16 risk factors examined (p-value < 0.003).

Table S5. Multivariable logistic regression models for screening for neurodevelopmental delay between before and during COVID-19 period among children aged 30–36 months according to the SES level (three types of insurance).

| The 7 domains of the K-DST | Medicaid | | Self-employed insured | | Insured employee | |
| --- | --- | --- | --- | --- | --- | --- |
|  | aOR | P-value | aOR | P-value | aOR | P-value |
| Any domain | 1.31(1.20, 1.43) | <0.0001* | 1.14(1.11, 1.17) | <0.0001* | 1.10(1.09, 1.11) | <0.0001* |
| Gross Motor | 1.10(0.97, 1.26) | 0.142 | 1.02(0.98, 1.07) | 0.300 | 0.93(0.91, 0.96) | <0.0001* |
| Fine Motor | 1.28(1.15, 1.44) | <0.0001* | 1.11(1.07, 1.15) | <0.0001* | 1.06(1.04, 1.08) | <0.0001* |
| Cognition | 1.23(1.10, 1.37) | 0.0003* | 1.14(1.10, 1.18) | <0.0001* | 1.08(1.06, 1.10) | <0.0001* |
| Communication | 1.41(1.28, 1.55) | <0.0001* | 1.23(1.20, 1.27) | <0.0001* | 1.20(1.18, 1.21) | <0.0001* |
| Social Interaction | 1.30(1.16, 1.45) | <0.0001* | 1.20(1.16, 1.24) | <0.0001* | 1.13(1.11, 1.15) | <0.0001* |
| Self-Care | 1.29(1.14, 1.45) | <0.0001* | 1.18(1.13, 1.22) | <0.0001* | 1.13(1.11, 1.15) | <0.0001* |

Adjusting for children’s sex, age(continuous), residence level, preterm birth or LBW, maternal age group, mother’s nationality, number of pediatricians per population of children, delayed visit, and section type of survey

* Significant after Bonferroni correction for 16 risk factors examined (p-value < 0.003).

Table S6. Multivariable logistic regression models for screening for neurodevelopmental delay between before and during COVID-19 period among children aged 30–36 months according to their residence.

|  |  |  |  | Screened for neurodevelopmental delay | | | |  | Interaction term | |
| --- | --- | --- | --- | --- | --- | --- | --- | --- | --- | --- |
| The 7 Domains of the K-DST |  | Urban | |  |  | Rural | |  |  |  |
|  | OR (95% CI) | P-value | aOR (95% CI) | P-value | OR (95% CI) | P-value | aOR (95% CI) | P-value | Wald Chi-Square | p-value |
| Any domain | 1.11(1.09, 1.13) | <0.0001* | 1.16(1.14, 1.18) | <0.0001* | 1.05(1.03, 1.06) | <0.0001* | 1.08(1.06, 1.09) | <0.0001* | 31.59 | <0.0001 |
| Gross Motor | 1.00(0.97, 1.03) | <0.0001* | 1.00(0.97, 1.04) | <0.0001* | 0.93(0.90, 0.95) | 0.910 | 0.92(0.90, 0.95) | 0.943 | 16.32 | <0.0001 |
| Fine Motor | 1.09(1.07, 1.12) | 0.010 | 1.12(1.10, 1.15) | 0.010 | 1.03(1.01, 1.05) | <0.0001* | 1.04(1.02, 1.07) | <0.0001* | 16.95 | <0.0001 |
| Cognition | 1.09(1.06, 1.12) | 0.001* | 1.14(1.11, 1.16) | 0.001* | 1.04(1.01, 1.06) | <0.0001* | 1.07(1.05, 1.09) | <0.0001* | 10.47 | 0.001 |
| Communication | 1.19(1.17, 1.22) | <0.0001* | 1.24(1.22, 1.27) | <0.0001* | 1.15(1.13, 1.17) | <0.0001* | 1.18(1.16, 1.20) | <0.0001* | 6.79 | 0.009 |
| Social Interaction | 1.16(1.14, 1.19) | <0.0001* | 1.18(1.15, 1.21) | <0.0001* | 1.13(1.10, 1.15) | <0.0001* | 1.13(1.11, 1.15) | <0.0001* | 5.33 | 0.021 |
| Self-Carel | 1.11(1.08, 1.13) | <0.0001* | 1.18(1.15, 1.21) | <0.0001* | 1.05(1.03, 1.07) | <0.0001* | 1.11(1.09, 1.13) | <0.0001* | 14.94 | 0.0001 |

Adjusting for children’s sex, age(continuous), socioeconomic status level, preterm birth or LBW, maternal age group, mother’s nationality, number of pediatricians per population of children, delayed visit, and section type of survey

* Significant after Bonferroni correction for 16 risk factors examined (p-value < 0.003).

Table S7. Multivariable logistic regression models for screening for neurodevelopmental delay between before and during COVID-19 period among children aged 30–36 months old.

| The 7 domains of the K-DST | Screened for neurodevelopmental delay | |
| --- | --- | --- |
|  | aOR | P-value |
| Any | 1.08(1.07, 1.10) | <.0001* |
| Gross Motor | 0.93(0.91, 0.95) | <.0001* |
| Fine Motor | 1.05(1.04, 1.07) | <.0001* |
| Cognition | 1.07(1.05, 1.09) | <.0001* |
| Communication | 1.18(1.16, 1.19) | <.0001* |
| Social Interaction | 1.13(1.11, 1.14) | <.0001* |
| Self-Care | 1.11(1.10, 1.13) | <.0001* |

Adjusting for children’s sex, age group, urban, socioeconomic status, preterm birth or LBW, maternal age group, mother’s nationality, number of pediatricians per population of children, delayed visit, and disability

* Significant after Bonferroni correction for 16 risk factors examined (p-value < 0.003).

Table S8. Multivariable logistic regression models for screening for neurodevelopmental delay in 30–36-month-old children: stratification by the disability status during the before and during COVID-19 periods.

| The 7 domains of the K-DST | Screened for the risk of neurodevelopmental delay | | | | |
| --- | --- | --- | --- | --- | --- |
|  | With disability | | | Without disability | |
|  | aOR | P-value | | aOR | P-value |
| Any | 1.08(0.92, 1.26) | | 0.356 | 1.11(1.10, 1.12) | <.0001* |
| Gross Motor | 0.99(0.85, 1.15) | | 0.882 | 0.96(0.94, 0.97) | <.0001* |
| Fine Motor | 1.10(0.94, 1.28) | | 0.226 | 1.08(1.06, 1.09) | <.0001* |
| Cognition | 1.11(0.95, 1.29) | | 0.182 | 1.10(1.08, 1.11) | <.0001* |
| Communication | 1.15(0.99, 1.34) | | 0.078 | 1.21(1.19, 1.22) | <.0001* |
| Social Interaction | 1.21(1.03, 1.40) | | 0.017 | 1.15(1.13, 1.17) | <.0001* |
| Self-Care | 1.13(0.97, 1.32) | | 0.117 | 1.14(1.12, 1.16) | <.0001* |

Adjusting for children’s sex, age group, urban, socioeconomic status, preterm birth or LBW, maternal age group, mother’s nationality, number of pediatricians per population of children, and delayed visit

* Significant after Bonferroni correction for 16 risk factors examined (p-value < 0.003).

Table S9. Multivariable logistic regression models assessing neurodevelopmental delay screening in 30–36-month-old children: stratification by preterm birth or low birth weight during before and during COVID-19 periods.

| The 7 domains of the K-DST | Screened for the risk of neurodevelopmental delay | | | | |
| --- | --- | --- | --- | --- | --- |
|  | Preterm or LBW | | | Normal birth | |
|  | aOR | P-value | | aOR | P-value |
| Any | 1.13(1.08, 1.17) | | <.0001* | 1.11(1.10, 1.12) | <0.0001* |
| Gross Motor | 1.02(0.95, 1.08) | | 0.6575 | 0.95(0.93, 0.97) | <0.0001* |
| Fine Motor | 1.10(1.04, 1.16) | | 0.0005* | 1.07(1.06, 1.09) | <0.0001* |
| Cognition | 1.17(1.10, 1.24) | | <0.0001* | 1.09(1.07, 1.11) | <0.0001* |
| Communication | 1.20(1.14, 1.25) | | <0.0001* | 1.21(1.19, 1.23) | <0.0001* |
| Social Interaction | 1.16(1.09, 1.22) | | <0.0001* | 1.15(1.13, 1.17) | <0.0001* |
| Self-Care | 1.17(1.11, 1.23) | | <0.0001* | 1.14(1.12, 1.15) | <0.0001* |

Adjusting for children’s sex, age group, urban, socioeconomic status, disability, maternal age group, mother’s nationality, number of pediatricians per population of children, and delayed visit

* Significant after Bonferroni correction for 16 risk factors examined (p-value < 0.003).

Table S10. Risk factors for neurodevelopmental delay. (Continued).

|  | Any | | Gross Motor | | Fine Motor | | Cognition | |
| --- | --- | --- | --- | --- | --- | --- | --- | --- |
|  | aOR  (95% CI) | P-value | aOR  (95% CI) | P-value | aOR  (95% CI) | P-value | aOR  (95% CI) | P-value |
| During the COVID-19 pandemic (ref. Pre) | 1.11(1.10, 1.12) | <0.0001 | 0.96(0.94, 0.97) | <0.0001 | 1.08(1.06, 1.09) | <0.0001 | 1.10(1.08, 1.11) | <0.0001 |
| Age(continuous) | 0.89(0.88, 0.89) | <0.0001 | 0.89(0.88, 0.90) | <0.0001 | 0.90(0.89, 0.91) | <0.0001 | 0.87(0.87, 0.88) | <0.0001 |
| Preterm birth or LBW |  |  |  |  |  |  |  |  |
| Yes | 1.34(1.31, 1.36) | <0.0001 | 1.53(1.48, 1.58) | <0.0001 | 1.40(1.36, 1.44) | <0.0001 | 1.44(1.40, 1.48) | <0.0001 |
| No | 1 |  | 1 |  | 1 |  | 1 |  |
| Sex |  |  |  |  |  |  |  |  |
| Boys | 2.16(2.14, 2.18) | <0.0001 | 1.35(1.33, 1.38) | <0.0001 | 2.34(2.30, 2.37) | <0.0001 | 1.95(1.92, 1.99) | <0.0001 |
| Girls | 1 |  | 1 |  | 1 |  | 1 |  |
| Types of the insured ^a^ |  |  |  |  |  |  |  |  |
| Medicaid | 1.83(1.75, 1.91) | <0.0001 | 2.18(2.04, 2.33) | <0.0001 | 1.97(1.86, 2.09) | <0.0001 | 2.46(2.32, 2.60) | <0.0001 |
| Non-Medicaid | 1 |  | 1 |  | 1 |  | 1 |  |
| Disability |  |  |  |  |  |  |  |  |
| Yes (Mild or Severe) | 7.93(7.36, 8.55) | <0.0001 | 18.01(16.74, 19.39) | <0.0001 | 10.52(9.76, 11.33) | <0.0001 | 14.27(13.25, 15.36) | <0.0001 |
| None |  |  | 1 |  | 1 |  | 1 |  |
| Number of pediatricians per population of children |  |  |  |  |  |  |  |  |
| None | 1.00(0.96, 1.03) | 0.003 | 1.13(1.05, 1.20) | 0.033 | 1.04(0.99, 1.10) | 0.783 | 1.11(1.05, 1.17) | 0.108 |
| Under mean | 1.11(1.10, 1.12) | <0.0001 | 1.10(1.08, 1.12) | 0.034 | 1.10(1.08, 1.12) | <0.0001 | 1.12(1.10, 1.14) | <0.0001 |
| Mean and over |  |  | 1 |  | 1 |  | 1 |  |
| Region |  |  |  |  |  |  |  |  |
| Rural | 0.95(0.94, 0.96) | <0.0001 | 0.96(0.94, 0.98) | <0.0001 | 0.94(0.93, 0.96) | <0.0001 | 1.03(1.01, 1.04) | 0.003 |
| Urban |  |  | 1 |  | 1 |  | 1 |  |
| Delayed visit |  |  |  |  |  |  |  |  |
| Yes | 1.09(1.07, 1.11) | <0.0001 | 1.14(1.11, 1.19) | <0.0001 | 1.06(1.04, 1.10) | <0.0001 | 1.15(1.11, 1.18) | <0.0001 |
| No | 1 |  | 1 |  | 1 |  | 1 |  |
| Section type of survey |  |  |  |  |  |  |  |  |
| For 36-41 months | 1.49(1.46, 1.53) | <0.0001 | 1.73(1.63, 1.82) | <0.0001 | 1.69(1.61, 1.76) | <0.0001 | 1.59(1.52, 1.66) | <0.0001 |
| For 33-35 months | 1.63(1.58, 1.67) | <0.0001 | 1.29(1.24, 1.34) | 0.120 | 1.61(1.56, 1.66) | <0.0001 | 1.53(1.48, 1.59) | <0.0001 |
| For 30-32 months | 1 |  | 1 |  | 1 |  | 1 |  |
| Mother’s age group |  |  |  |  |  |  |  |  |
| 20-29 | 1.11(1.03, 1.19) | <0.0001 | 1.08(0.96, 1.20) | <0.0001 | 1.14(1.04, 1.25) | <0.0001 | 1.10(1.01, 1.20) | <0.0001 |
| 30-39 | 0.85(0.79, 0.91) | <0.0001 | 0.83(0.74, 0.92) | <0.0001 | 0.84(0.76, 0.92) | <0.0001 | 0.72(0.66, 0.78) | <0.0001 |
| 40 and over | 0.93(0.86, 0.99) | <0.0001 | 0.86(0.77, 0.96) | <0.0001 | 0.86(0.79, 0.95) | <0.0001 | 0.84(0.77, 0.92) | <0.0001 |
| Unknown | 1 |  | 1 |  | 1 |  | 1 |  |
| Mother’s nationality |  |  |  |  |  |  |  |  |
| Korea | 0.54(0.52, 0.55) | <0.0001 | 0.52(0.49, 0.54) | <0.0001 | 0.57(0.55, 0.59) | <0.0001 | 0.45(0.44, 0.47) | <0.0001 |
| Foreign | 1 |  | 1 |  | 1 |  | 1 |  |
| Unknown | 0.43(0.4, 0.45) | <0.0001 | 0.43(0.39, 0.48) | <0.0001 | 0.46(0.42, 0.50) | <0.0001 | 0.28(0.25, 0.30) | <0.0001 |

a Insured type of parents or legal guardians. Non-Medicaid group indicated the employee insured or the self-employed insured.

Table S10. Risk factors for neurodevelopmental delay..

|  | Communication | | Social Interaction | | Self-Control | |
| --- | --- | --- | --- | --- | --- | --- |
|  | aOR  (95% CI) | P-value | aOR  (95% CI) | P-value | aOR  (95% CI) | P-value |
| During the COVID-19 pandemic (ref. Pre) | 1.21(1.19, 1.22) | <0.0001 | 1.15(1.13, 1.17) | <0.0001 | 1.14(1.12, 1.16) | <0.0001 |
| Age(continuous) | 0.88(0.87, 0.88) | <0.0001 | 0.90(0.90, 0.91) | <0.0001 | 0.88(0.87, 0.88) | <0.0001 |
| Preterm birth or LBW |  |  |  |  |  |  |
| Yes | 1.40(1.37, 1.44) | <0.0001 | 1.33(1.29, 1.36) | <0.0001 | 1.32(1.28, 1.35) | <0.0001 |
| No | 1 |  | 1 |  | 1 |  |
| Sex |  |  |  |  |  |  |
| Boys | 2.36(2.33, 2.39) | <0.0001 | 2.30(2.27, 2.34) | <0.0001 | 2.91(2.86, 2.96) | <0.0001 |
| Girls | 1 |  | 1 |  | 1 |  |
| Types of the insured ^a^ |  |  |  |  |  |  |
| Medicaid | 2.30(2.19, 2.41) | <0.0001 | 2.16(2.04, 2.28) | <0.0001 | 1.55(1.46, 1.64) | <0.0001 |
| Non-Medicaid | 1 |  | 1 |  | 1 |  |
| Disability |  |  |  |  |  |  |
| Yes (Mild or Severe) | 10.57(9.82, 11.39) | <0.0001 | 12.56(11.66, 13.52) | <0.0001 | 10.34(9.59, 11.14) | <0.0001 |
| None | 1 |  | 1 |  | 1 |  |
| Number of pediatricians per population of children |  |  |  |  |  |  |
| None | 1.05(0.99, 1.09) | 0.858 | 1.02(0.97, 1.08) | 0.280 | 0.93(0.88, 0.98) | <0.0001 |
| Under mean | 1.10(1.09, 1.12) | <0.0001 | 1.10(1.09, 1.12) | <0.0001 | 1.08(1.07, 1.10) | <0.0001 |
| Mean and over | 1 |  | 1 |  | 1 |  |
| Region |  |  |  |  |  |  |
| Rural | 1.01(0.99, 1.02) | 0.359 | 0.95(0.94, 0.97) | <0.0001 | 0.91(0.90, 0.93) | <0.0001 |
| Urban | 1 |  | 1 |  | 1 |  |
| Delayed visit |  |  |  |  |  |  |
| Yes | 1.11(1.09, 1.14) | <0.0001 | 1.13(1.10, 1.16) | <0.0001 | 1.15(1.11, 1.18) | <0.0001 |
| No | 1 |  | 1 |  | 1 |  |
| Section type of survey |  |  |  |  |  |  |
| For 36-41 months | 1.28(1.24, 1.32) | 0.141 | 1.41(1.37, 1.46) | <0.0001 | 1.77(1.72, 1.83) | <0.0001 |
| For 33-35 months | 1.59(1.54, 1.66) | <0.0001 | 1.63(1.56, 1.70) | <0.0001 | 1.39(1.33, 1.45) | 0.0123 |
| For 30-32 months | 1 |  | 1 |  | 1 |  |
| Mother’s age group |  |  |  |  |  |  |
| 20-29 | 1.20(1.11, 1.29) | <0.0001 | 1.13(1.03, 1.23) | <0.0001 | 0.99(0.90, 1.09) | 0.040 |
| 30-39 | 0.83(0.77, 0.90) | <0.0001 | 0.85(0.78, 0.93) | <0.0001 | 0.87(0.80, 0.96) | <0.0001 |
| 40 and over | 1.02(0.95, 1.11) | 0.140 | 1.02(0.94, 1.11) | 0.073 | 0.97(0.88, 1.07) | 0.300 |
| Unknown | 1 |  | 1 |  | 1 |  |
| Mother’s nationality |  |  |  |  |  |  |
| Korea | 0.41(0.40, 0.42) | <0.0001 | 0.42(0.40, 0.43) | <0.0001 | 0.57(0.55, 0.59) | <0.0001 |
| Foreign | 1 |  | 1 |  | 1 |  |
| Unknown | 0.30(0.28, 0.32) | <0.0001 | 0.33(0.31, 0.36) | <0.0001 | 0.50(0.45, 0.54) | <0.0001 |

a Insured type of parents or legal guardians. Non-Medicaid group indicated the employee insured or the self-employed insured.

Figure S1. Annual number of infants and the rate (%) of follow up based on birth statistics by the National Health Insurance Service databases among 30-36 months old.


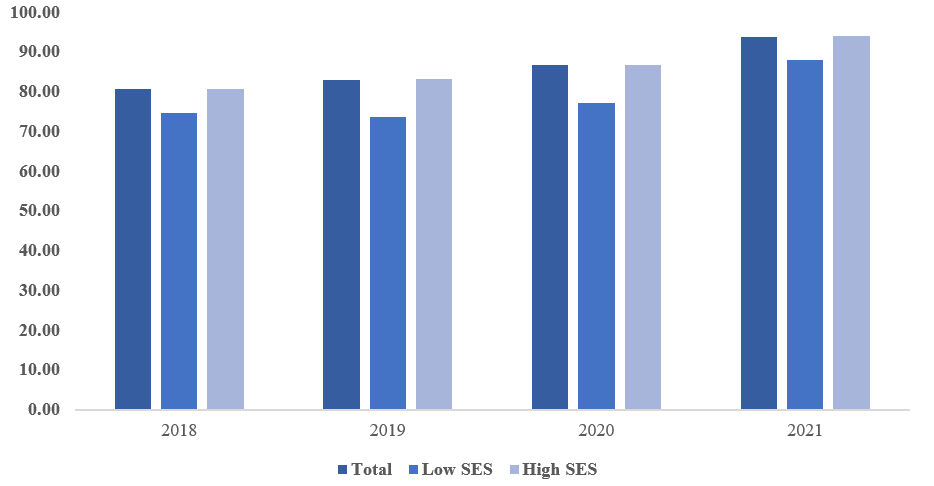


Low SES group indicated the group of Medicaid. High SES group indicated non-Medicaid group including the employee insured or the self-employed insured.
